# Intestinal epithelial TLR4 knock out induces sex-specific effects on gut barrier and microbiome in an activity-based anorexia model

**DOI:** 10.1080/19490976.2026.2637316

**Published:** 2026-02-28

**Authors:** Colin Salaün, Marion Huré, Charlène Guérin, Christine Bôle-Feysot, Audrey Valentin, Fatima Léon, Sarah Lenoir, Jean-Luc do-Rego, Jean-Claude do-Rego, Ludovic Langlois, David Ribet, Najate Achamrah, Moïse Coëffier

**Affiliations:** a INSERM, Normandie Université, ADEN UMR1073 'Nutrition, Inflammation and microbiota-gut-brain axis', Univ Rouen Normandie, Rouen, France; b Institute for research and Innovation in Biomedicine (IRIB), Univ Rouen Normandie, Rouen, France; c INSERM, CNRS, HeRacLeS US51 UAR2026, Animal Behavioural Platform, SCAC, Univ Rouen Normandie, Rouen, France; d Department of Nutrition and CIC-CRB 1404, CHU Rouen, Rouen, France

**Keywords:** Anorexia nervosa, activity-based anorexia mice, gut microbiota, gut barrier, gut inflammation, TLR4

## Abstract

The role of the microbiota‒gut‒brain axis in the pathophysiology of anorexia nervosa has emerged in recent decades. Increased expression of Toll-like receptor 4 (TLR4) has been reported in the intestinal epithelial cells (IEC) of activity-based anorexia (ABA) mice. The inducible TLR4 knockout in IEC (TLR4^IEC^^−/−^) was subsequently associated with behavioral and energy balance changes in ABA mice. Our study aimed to assess the intestinal response to TLR4^IEC^^−/−^ in both male and female ABA mice by focusing on three components: inflammation, the gut barrier, and the gut microbiota composition. After 12 d of undernutrition with free wheel access, the colonic expression of 43 markers was measured by RT-qPCR. The gut microbiota composition was analyzed by Illumina sequencing of the 16S rRNA gene. First, TLR4^IEC^^−/−^ was associated with more marked alterations in male control mice compared to females. Indeed, a reduction in the mRNA expression of eight inflammatory factors, seven tight junction proteins and fecal calprotectin levels was observed in males. Control TLR4^IEC^^−/−^ females showed increased expression of four inflammatory markers and one target involved in the gut barrier. The levels of the *Bacillota* phylum and the *Deltaproteobacteria* class and their subdivisions, up to the *Desulfovibrio* genus, increased in the control TLR4^IEC^^−/−^ males compared to wt. In females, only an increase in the *Alcaligenaceae* genus, which ranks from the *Betaproteobacteria* phylum, was observed. Interestingly, in both males and females, these alterations were not observed in response to ABA model in TLR4^IEC^^−/−^ mice. Similarly, ABA increased *Tjp1* expression and *Lactobacillus* abundance, both of which were decreased by TLR4^IEC^^−/−^. Our study shows for the first time the impact of inducible TLR4^IEC^^−/−^ on the intestinal response. TLR4^IEC^^−/−^ induced sex-specific colonic alterations and changes in the gut microbiota, which disappeared after the ABA model. Further studies are warranted to decipher the underlying mechanisms.

## Graphical abstract

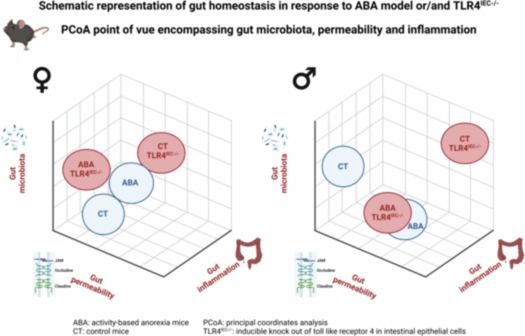

## Introduction

Anorexia nervosa (AN) is a severe eating disorder characterized by an abnormally low body weight (body mass index < 18.5 kg·m²), an intense fear of gaining weight and a dysmorphophobia according to the Diagnostic and Statistical Manual of Mental Disorders (DSM-5).^1^ The prevalence of AN is increasing. The literature suggests that approximately 1.4% of women and 0.2% of men will develop AN during their lifetime.^2^ AN also has the highest mortality rate among psychiatric disorders. In a 5-y follow-up study, 227 out of 5169 women with AN died, resulting in a 4.4% mortality rate.^3^ Patients suffering from AN often exhibit compensatory behaviors. Indeed, from 30% to 80% of individuals with AN have inappropriate exercise patterns.^4^^,^^5^ Interestingly, the activity-based anorexia (ABA) model is commonly employed to study AN and shows numerous pathophysiological features observed in patients.^6^^,^^7^ For instance, altered physical activity patterns,^5^^,^^8^ altered brain functions and behaviors,^9^^,^^10^ intestinal hyperpermeability and inflammatory markers^11-13^ have been described both in ABA mice and in patients with AN.

Previous studies highlighted the role of the microbiota‒gut–brain axis in the regulation of food-related behavior and in the pathophysiology of eating disorders, particularly in AN.^14^^,^^15^ In ABA mice, a disrupted gut barrier function and increased intestinal inflammation have been reported. Indeed, both TNFα and IL-1β mRNAs were increased in female ABA mice. In addition, Toll-like receptor 4 (TLR4) mRNA expression was reported to be increased in the colonic mucosa of female ABA mice, with a particularly high density of this protein in the gut epithelium.^11^ TLR4 essentially recognized the lipopolysaccharide (LPS) of bacteria, especially present in gram-negative, and is known for its effects on the regulation of food intake. Notably, after infection,^16^ it mediates the anorexigenic effects of LPS, but its role in feeding behavior in AN remains poorly documented. Surprisingly, TLR4 knockout (KO) mice exhibited a high rate of mortality in response to the ABA model.^11^ In contrast, specific KO of TLR4 in intestinal epithelial cells (TLR4^IEC^^−/−^) induced beneficial effects on behavior and/or energy homeostasis but in a sex-dependent manner.^10^ In this preliminary study, male ABA TLR4^IEC^^−/−^ mice exhibited a limited body weight loss during the initial days of undernutrition, although this effect did not reach significance in females. In addition, while wild-type (*wt*) male ABA mice exhibited higher corticosterone levels and increased immobility time in the behavioral test compared to controls. This effect disappeared when ABA TLR4^IEC^^−/−^ mice were compared with CT TLR4^IEC^^−/−^ mice.^10^ Although the impact of TLR4 depletion in IEC on feeding behavior and anxiety responses has been studied, its consequences on intestinal responses in ABA mice remain unknown.

In the present study, we thus aimed to get deeper insights into the effects of intestinal epithelial TLR4 KO during the ABA model on behavior and energy homeostasis and to evaluate its impact on the intestinal inflammatory response and gut barrier function both in female and male mice.

## Material and method

### Animal experimentation

The present project was approved by the regional ethical committee for animal experimentation (CENOMEXA APAFIS#36881-2022042014271193 v4), and we obtained the authorization to use TLR4 genetically modified rodent models (DUO8167). For all the experiments, female and male mice were fed a standard diet A03-3430 (SAFE) and housed at the platform for animal behavior (‘Service Commun d'Analyze Comportementale’ or SCAC, HeRacLeS unit, Rouen University) at 21 °C with inversed light/dark cycle.

### KO of TLR4 in intestinal epithelial cells

We crossed Villin-Cre^ERT2^ mice (a kind gift from Dr. Sylvie Robine team, Institut Curie, Paris, France) and TLR4_floxed_ mice (Jackson Laboratory, US) to obtain Villin-Cre^ERT2^ TLR4_floxed_ mice. Then, we invalidated the TLR4 specifically in IEC in Villin-Cre^ERT2^ TLR4_floxed_ mice through injections of tamoxifen (TMX; T5648, Sigma). TMX intraperitoneal injections were administered in nine weeks old female and 12 weeks old male mice through five daily injections of 1 mg dissolved in a solution of 10% ethanol (VWR) and 90% sunflower oil, as previously described.^17^ Control mice were injected with PBS. We selected mice homozygous for the floxed TLR4 and expressing Cre recombinase.

The genotype of the mice was checked both after birth and postmortem as previously described.^10^ As the second LoxP position remained unknown, we performed *Tlr4* gene sequencing after excision induced by Cre recombinase to localize LoxP sites (Figure S1A–S1D). For few mice, Cre recombinase activity was not detected. These mice were thus excluded from the analyzes. As a result, two CT males, two ABA males, and one ABA female were removed from the study.

### Activity-based anorexia model

**Figure 1.**
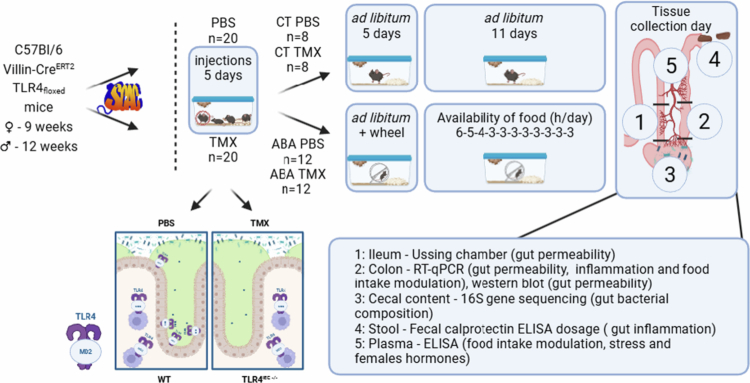
Experimental design. Females and males Villin-Cre^ERT2^ TLR4_floxed_, KO or not for the TLR4 in intestinal epithelial cells (IEC) were submitted to the activity-based anorexia (ABA) model or not (CT) before tissue sampling and analyzes.

After TMX injections, the mice were subjected to the ABA protocol, or if not, were housed in standard cages (CTs). ABA experiments were conducted as previously described.^18^ Briefly, the mice were housed in cages equipped with running wheels (Intellibio, Seichamps, France; Activiwheel software). After a period of acclimatization to the cages with free access to food (day 1–day 5), a progressive limitation of access to food was induced according to the following schedule: 6–5–4–3–3–3–3–3–3–3–3 hours per day from day 6 to day 16, respectively. Food was given at the beginning of the dark phase. During the protocol, body weight, food, and water intake were monitored daily at the end of the light phase (Figure 1).

At the end of the experiments (day 16), the mice were anaesthetized intraperitoneally with ketamine (Boehringer Ingelheim, COVETO, Caen, France; 100 mg/kg) and xylazine (Bayer HealthCare, Puteaux, France; 10 mg/kg) diluted in 0.9% NaCl at the end of the resting period (10 µL/g). Blood was then collected from the inferior vena cava in heparin-coated Vacutainer tubes (BD-Plymouth, UK). The plasma was subsequently obtained by centrifugation at 3000 × *g* for 15 min at 4 °C. Ileum segments were mounted in a Ussing chamber, and colon segments and cecal content were snap frozen in liquid nitrogen and stored at −80 °C until nucleic acids or proteins extraction (Figure 1).

### Immunoassays on plasma and fecal samples

Gut peptides regulating metabolism and food intake, GLP1 and PYY (Phoenix Pharmaceuticals, Karlsruhe, Germany), corticosterone (Abnova, KA0468, VWR international SAS, Fontenay-sous-Bois, France), estradiol E2 (Cusabio, CSB-E05109m), and progesterone (antibodies online, ABIN6969565) were measured by enzyme immunoassays in plasma samples (Figure 1).

At the end of ABA experiments, feces were collected and immediately stored at −80 °C until protein extraction. Then, fecal calprotectin, a marker of intestinal inflammation, was quantified by ELIZA (S100A8/S100A, R&D System, Minneapolis, US) following the manufacturer's recommendations as previously described.^19^

### Paracellular ileal permeability

Ileal segments were placed in Ussing chambers (Harvard Apparatus, Holliston, MA, US), and the paracellular permeability was evaluated by measuring the flux of FITC-dextran (4 kDa; excitation: 485 nm; emission: 535 nm) from the mucosal to the serosal sides after 3 h of incubation, as previously described.^13^

### RT-qPCR

Harvested colons were immediately frozen in liquid nitrogen and stored at −80 °C until RNA extraction to perform RT-qPCR on inflammation markers and gut barrier genes. Total RNA was extracted from the colon using the TRIzol–Chloroform (Invitrogen, Carlsbad, CA, USA; Merck, Darmstadt, Germany) based on the method described by Chomczynski and Sacchi.^20^ The quality and integrity of the RNAs were assessed via agarose gel electrophoresis. The RNA concentrations were quantified using a Nanodrop 2000 spectrophotometer (Thermo Fisher Scientific, Illkirch, France). RNA samples were then treated with DNase (Promega, Charbonnières-les-Bains, France) to remove any contaminating genomic DNA, followed by reverse transcription using M-MLV Reverse Transcriptase (Invitrogen) to generate cDNA as previously described.^18^

qPCR targeting 43 markers of interest mainly involved in inflammatory responses (*Nod2*, *Tlr2*, *Tlr4*, *Cd14*, *Ticam1*, *Irf3*, *Myd88*, *Nfκb*, *Nfκb-iα*, *Tnfα*, *Il1β*, *Il4, Il6, Il10, Ifng*, *Tgfβ*, *Cxcl1*, *Ccl2,* and *Cxcr3*), gut barrier functions (*Ocln*, *Marveld2*, *F11r*, *Igsf5*, *Cgn*, *Tjp1*, *Tjp2*, *Tjp3*, *Claudins 1*, *2*, *3*, *4*, *5*, *7*, *8*, *11*, *12,15*, and *Muc2*), and eating behavior regulation (*Gcg*, *Yy* peptide) were then performed using the SYBR Green technology on a QuantStudio 12K Flex real-time PCR system (Life Technologies, Carlsbad, CA, US) at PRIMCACEN platform (HeRacLeS US51, Rouen University; Figure 1). cDNA levels were normalized using the mean of the housekeeping genes *Actb*, *Gapdh,* and *B2m.* The sequences and melting temperatures of all the oligonucleotides used in the study are detailed as reported^21^ except for *Muc2* (F: ATGACGTCTGGTGGAATGGT; R: TGTTCTGACAGTTGCACGTG; 60 °C).

### Western blotting

Western blots were performed on the colon to evaluate tight junction markers following two protein extraction method. The first lysis buffer used for the study of ZO-1 (Tjp-1) and Occludin was composed, as previously described,^22^ of 25 mM spermine tetrahydrochloride, protease, and phosphatase inhibitors (all from Sigma-Aldrich, St. Louis, US), 50 mM DTT, 4% CHAPS (both Janssen Pharmaceuticalaan, Geel, Belgium), and 8 M urea (Merck Millipore, Burlington, US). After mechanical lysis and centrifugation, the supernatants were obtained, and protein concentrations were assessed using 2D quant kit (Cytiva, Marlborough, US). The second permitted the study of Claudin-1 as formerly described: Hepes pH 7.9 10 mM, KCl 10 mM, MgCl_2_ 1.5 mM, EDTA 0.1 mM, DTT 1 mM, NP40 0.25%, protease inhibitor 0.5%, and phosphatase inhibitor 1%.^23^

A denaturing electrophoresis was used to separate proteins on stain-free polyacrylamide gels (Mini-PROTEAN TGX Stain-Free Gels; Bio-Rad Laboratories, Marnes la Coquette, France). Proteins were then transferred onto nitrocellulose (GE Healthcare) or PVDF (Bio-Rad Laboratories) for Claudin-1 or for Occludin and ZO-1, respectively. After blockage for 1 h in 5% milk, the membranes were incubated overnight at 4 °C with primary antibody (Claudin-1 (71-7800, Thermo Fisher Scientific, Waltham, MA, US), Occludin (33-1500, Thermo Fisher Scientific), and ZO-1 (33-9100, Thermo Fisher Scientific) at 1/1000^e^). After being washed with 0.2% TBS-T, the membranes were incubated with the adapted secondary antibody (1/5000; DakoCytomation, Denmark). Other washes were followed by chemiluminescence revelation by using Clarity kit (Bio-Rad Laboratories).

Data acquisition and analysis were performed using a ChemiDoc™ XRS+ and Image Lab™ software (Bio-Rad Laboratories). The results are expressed by rationalizing the intensity of the bands of interest to that of the stain-free total proteins.

### Gut microbiota 16S rRNA analyzes

Cecal contents were immediately frozen in liquid nitrogen and stored at −80 °C until DNA extraction for 16S Illumina sequencing (Figure 1), which was subsequently analyzed using the EasyMAP online platform. Specifically, the QIAamp Fast DNA Stool Mini Kit (QIAGEN) was employed for extraction, as previously described.^24^ Subsequently, the DNA samples were sent to the University of Minnesota Genomics Center (UMGC) for Illumina MiSeq sequencing of the V5–V6 region of the 16S rRNA gene, resulting in the generation of 2 × 300 bp sequencing products. The obtained data were then analyzed according to the EasyMAP recommendations (http://easymap.cgm.ntu.edu.tw/).^25^ Pair-end filtering was conducted using the DADA2 plugin in QIIME-2 to obtain amplicon sequence variants (ASVs) based on the quality-filtered sequences, with a forward trimming range of 6–300 bp and a reverse trimming range of 20–300 bp. Next, the Silva database, which includes the V5–V6 region, was employed for taxonomic analysis.

### Impact of TMX on feeding behavior in wild-type mice

To evaluate the effects of TMX itself, we performed TMX or PBS injections into both female and male 8-week-old C57Bl/6 wild-type mice (Janvier Labs, Le Genest-Saint-Isle, France). Feeding behavior was evaluated by placing mice (female PBS *n* = 6; male PBS *n* = 6; female TMX *n* = 10; and male TMX *n* = 10) in BioDAQ food and drink intake monitor (BioDAQ, Research Diet, Inc., New Brunswick, NJ, US). The same TMX injection procedure as written above was used.

### Statistical analysis

**Figure 2.**
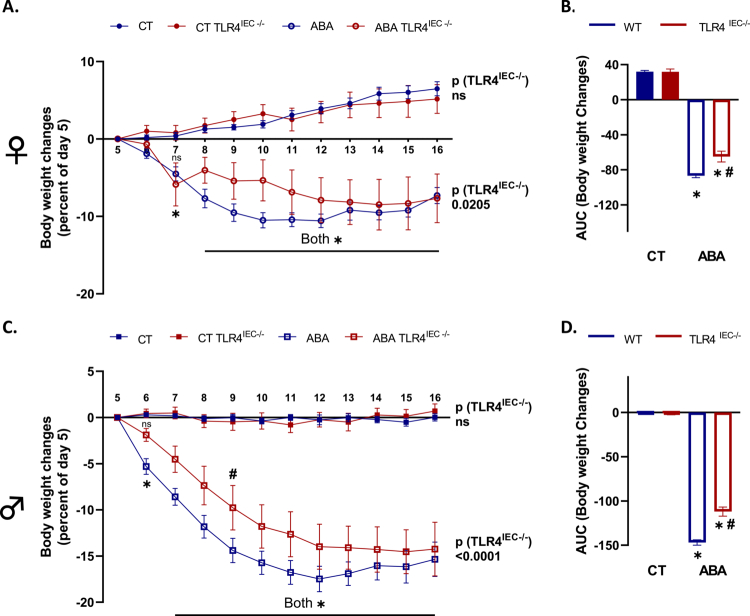
Body weight changes in female and male TLR4^IEC^^−/−^ mice in response to the ABA model. Body weight changes in female (A, B) and male (C, D) wild-type (*wt*) mice (in blue) and mice invalidated for TLR4 specifically in intestinal epithelial cells (IEC) (TLR4^IEC^^−/−^, in red) under control conditions (CT, closed symbols) or submitted to the activity-based anorexia (ABA) model (open symbols). (A and C) Data are shown as mean ± SEM and were analyzed using two-way ANOVA (TLR4^IEC^^−/−^ × time). The *p-*value (TLR4^IEC^^−/−^) is indicated for each condition. Bonferroni's multiple comparisons test is indicated as *p < 0.05 for ABA vs. respective CT and ^#^*p* < 0.05 for TLR4^IEC^^−/−^ vs. *wt* (*n* = 10–16 per group). (B and D) Area under the curve of body weight changes was analyzed with *t*-test for females and males. *p < 0.05 for ABA vs. respective CT and ^#^*p* < 0.05 TLR4^IEC^^−/−^ vs. *wt*.

**Figure 3.**
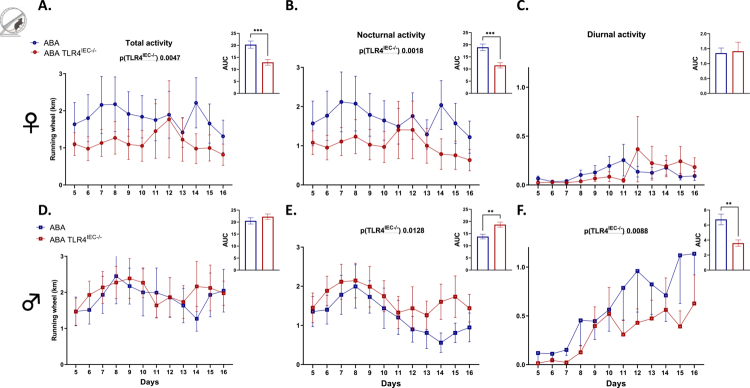
Running wheel activity in female and male TLR4^IEC^^−/−^mice in response to the ABA model. Running wheel activities in female (A, B, and C) and male (D, E, and F) wild-type (*wt*) mice (in blue) and mice invalidated for the TLR4 specifically in intestinal epithelial cells (TLR4^IEC^^−/−^, in red) submitted to the activity-based anorexia (ABA) model. Running wheel activity was analyzed as total physical activity (A and D), nocturnal physical activity (B and E), and diurnal physical activity (C and F). The data are shown as mean ± SEM and were analyzed using two-way ANOVA (TLR4^IEC^^−/−^ × time). The significant *p-*value (TLR4^IEC^^−/−^) is indicated in each graph. The area under the curve is shown in each panel with **p < 0.01 and ***p < 0.001 (Mann‒Whitney test, *n* = 10‒12 per group).

**Figure 4.**
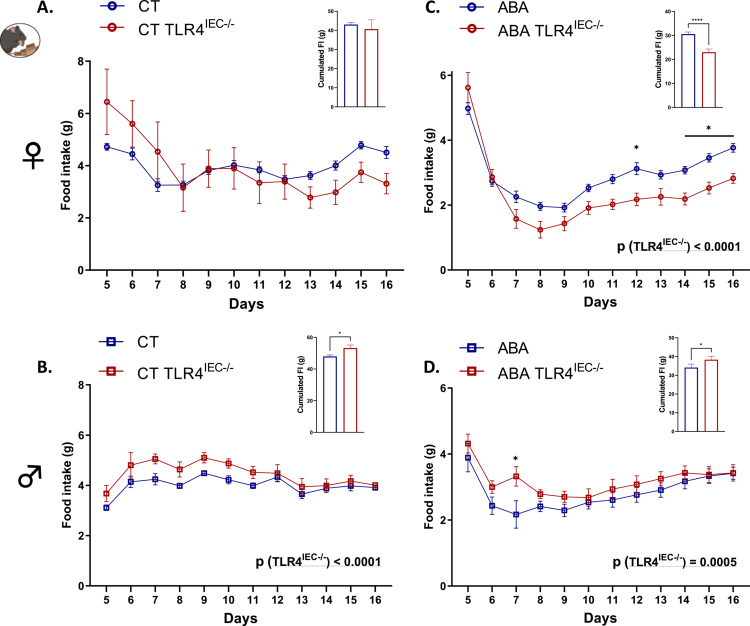
Food intake in female and male TLR4^IEC^^−/−^mice in response to the ABA model. Food intake (g) in female (A, C) and male (B, D) wild-type (*wt*) mice (in blue) and mice invalidated for the TLR4 specifically in intestinal epithelial cells (TLR4^IEC^^−/−^, in red) submitted to the activity-based anorexia (ABA) model (C, D) or under control conditions (CT; A, B). The data are shown as mean ± SEM and were analyzed using two-way ANOVA (TLR4^IEC^^−/−^ × time). The significant *p-*value (TLR4^IEC^^−/−^) is indicated in each graph. Šídák's multiple comparisons test is indicated as *p < 0.05 for TLR4^IEC^^−/−^ vs. *wt* (*n* = 10‒16 per group). Cumulated food intake is shown in each panel with *p < 0.05 and ****p < 0.0001 (Mann‒Whitney test).

All the statistical analyzes and graphs were performed using GraphPad Prism 9 (San Diego, USA). All graphs are presented as mean ± SEM on plots. Significant results were considered when *p* < 0.05. The kinetics of body weight, running wheel, and food intake were analyzed by two-way ANOVA (TLR4^IEC^^−/−^ × time) followed by multiple comparison tests. More specifically, for the body weight curve, Bonferroni's correction was used to compare the differences between the two, CT vs. ABA and *wt* vs. TLR4^IEC^^−/−^ as used in the same model.^10^ For running wheel and food intake, Šídák's post-tests were used to compare the TLR4^IEC^^−/−^ effect at the same time. Comparison of area under the curve of body weight (Figure 2) was performed by unpaired *t*-test. Comparison of area under the curve of the running wheel (Figure 3), cumulative food intake (Figure 4), food intake parameters (Figure S2), and ileal permeability (Figure 6) were analyzed by Mann–Whitney test. Grouped analysis of immunoassay and RT-qPCR data was performed by two-way ANOVA (ABA × TLR4^IEC^^−/−^), followed by Tukey's post hoc tests. For these, *p-*values and *n*-values, which can differ from those of mice *n* due to experimental troubleshooting, were indicated on an Excel table (Figure S10). The Kruskal‒Wallis test followed by Dunn's post-tests was used to evaluate TMX effects. Fecal calprotectin quantification was performed in duplicate, and analyzed using a nested *t*-test.

For gut microbiota analysis, alpha and beta diversity indices were calculated in accordance with the recommendations set forth by the EasyMAP pipeline using QIIME-2. Taxonomy differential abundance analyzes were conducted using the LefSe LDA pipeline, with Kruskal‒Wallis and Wilcoxon test significant thresholds set at *p* < 0.05 and a threshold on the logarithmic LDA score for discriminative features set at 2. The results were represented graphically as cladograms and bar charts. Kruskal‒Wallis pairwise comparisons were employed to analyze alpha diversity indices and PERMANOVA for Jaccard and unweighted UniFrac distances.

## Results

### The KO of TLR4 in intestinal epithelial cells affects energy homeostasis in response to the activity-based anorexia model in a sex-dependent manner

**Figure 5.**
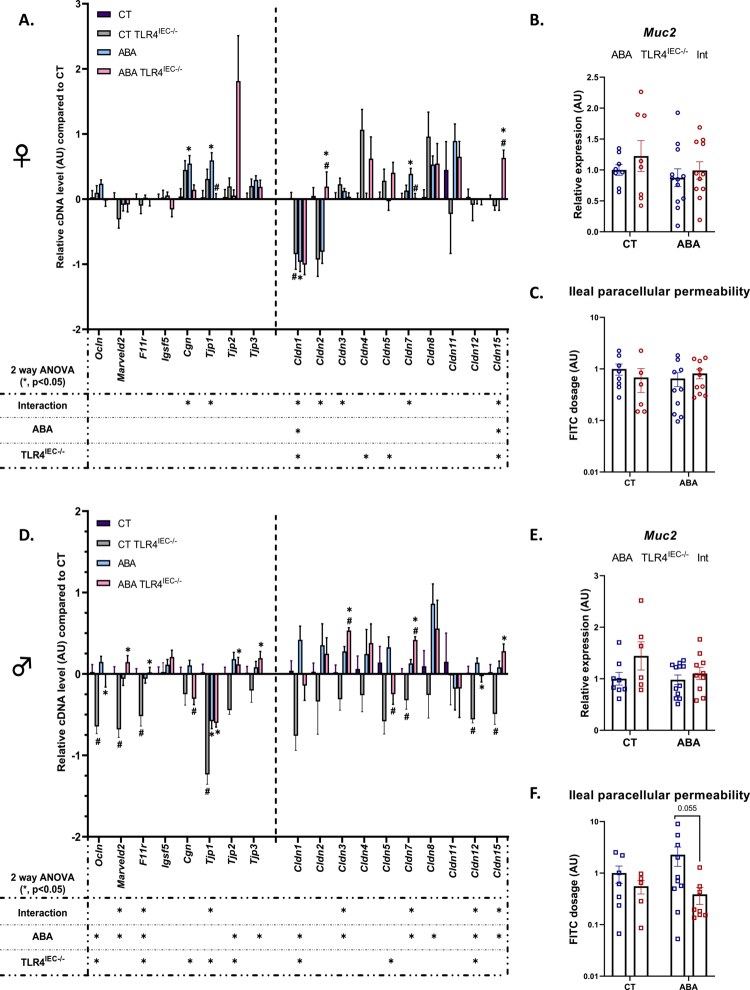
Gut permeability in female and male TLR4^IEC^^−/−^ mice in response to the ABA model. (A, B, D, and E): colonic tight junction protein markers and *Muc2* evaluated by RT-qPCR. Relative cDNA levels of tight junction protein markers and *Muc2* in the control (CT) TLR4^IEC^^−/−^, activity-based anorexia (ABA) and ABA TLR4^IEC^^−/−^ groups normalized to wild-type (*wt*) CT group in the proximal colon of female (A) and male (B) animals. *wt* CT mice or mice submitted to the activity-based anorexia (ABA) model were compared to mice invalidated for the TLR4 specifically in intestinal epithelial cells (TLR4^IEC^^−/−^). The data are shown as mean ± SEM bar plot and were analyzed using two-way ANOVA (TLR4^IEC^^−/−^ × ABA). The significance (*p < 0.05) were indicated just below the graph. Tukey's multiple comparisons tests are indicated as * (CT vs. ABA; CT TLR4^IEC^^−/−^vs. ABA TLR4^IEC^^−/−^) or # (CT vs. CT TLR4^IEC^^−/−^; ABA vs. ABA TLR4^IEC^^−/−^). *n* and exact *p-*value on Figure S10. (C and F): ileal paracellular permeability evaluated by Ussing chamber. The passage of fluorescent 4 kDa FITC-dextran molecules from the luminal to the mucosal compartment was evaluated by spectrophotometry on distal ileum segment, and expressed in comparison to the mean of the female control (CT). Wild-type (*wt*) mice and mice invalidated for the TLR4 specifically in intestinal epithelial cells (TLR4^IEC^^−/−^), were submitted to the activity-based anorexia (ABA) model or used under control conditions (CTs). Data are shown as mean ± SEM and were analyzed using Mann–Whitney test (*n* = 5‒10 per group).

As previously described,^10^ both female and male *wt* ABA mice exhibited a body weight loss at day 16 from day 5 (−7.3% and −15.3%, respectively, Figure 2A‒C). In control conditions, body weight changes were not affected by TLR4 KO in IEC, both in females (Figure 2A and B) and males (Figure 2C and D). In contrast, body weight loss was differentially modified in TLR4^IEC^^−/−^ ABA mice according to sex (both *p*(TLR4^IEC^^−/−^) < 0.05). Indeed, female TLR4^IEC^^−/−^ ABA mice showed a lower body weight loss from day 8 to day 12 compared to *wt* ABA mice (up to 5.2% at day 10, Figure 2A) while male TLR4^IEC^^−/−^ ABA mice exhibited a lower body weight loss from day 6 to day 13 (up to 4.6% at day 9, *p* < 0.05, Figure 2C). To better understand these responses, we analyzed both physical activities and energy intake in ABA mice. The physical activity pattern of ABA mice also showed differences related to TLR4 KO and sex (Figure 3). Female TLR4^IEC^^−/−^ ABA mice presented a lower total and nocturnal physical activity (both *p*(TLR4^IEC^^−/−^) <0.05, Figure 3A and B) compared to *wt* ABA mice, without impact on diurnal activity encompassing food anticipatory activity (Figure 3C). In contrast, total physical activity was not impaired in male TLR4^IEC^^−/−^ ABA mice compared to *wt* ABA mice (Figure 3D), while nocturnal and diurnal physical activities were, respectively, increased (*p*(TLR4^IEC^^−/−^) = 0.0128, Figure 3E) and decreased (*p*(TLR4^IEC^^−/−^) = 0.0088, Figure 3F). Concerning food intake, again, female and male mice exhibit different behaviors. While female TLR4^IEC^^−/−^ ABA mice presented a lower food intake compared to female *wt* ABA mice (*p*(TLR4^IEC^^−/−^) < 0.0001, Figure 4C), male TLR4^IEC^^−/−^ ABA mice showed a slight increase in food intake compared to male *wt* ABA mice (*p*(TLR4^IEC^^−/−^) = 0.0005, Figure 4D). These differences were also observed in cumulative food intake (Figure 4C and D; *p* < 0.0001 for females and *p* < 0.05 for males). Of note, food intake was not impacted by TLR4 KO in IECs from female CT mice (Figure 4A), whereas a slight increase was observed in males (*p*(TLR4^IEC^^−/−^) <0.0001, Figure 4B). As food intake was sex-dependently affected by TLR4 KO in IEC, we evaluated the intestinal endocrine response by assessing the mRNA expression and plasma concentration of GLP1 and PYY peptides (encoded by the *Gcg* and *Pyy* genes, respectively). In females, the ABA model induced an increase in colonic *Gcg* mRNA expression (*p*(ABA) = 0.0012, Figure 5A), particularly in TLR4^IEC^^−/−^ ABA mice (Tukey's post-test, *p* < 0.05). For *Pyy* mRNA levels, an interaction effect was observed without significant differences in post-tests. In contrast to these changes in gene expression, both plasma GLP1 and PYY levels remained unchanged in female *wt* and TLR4^IEC^^−/−^ ABA mice compared to their respective controls (Figure 5C). In males, TLR4 KO in IEC was associated with a lower *Gcg* mRNA level both in CT or ABA conditions (*p*(TLR4^IEC^^−/−^) = 0.0064, Figure 5B). No difference was observed for PYY mRNA expression. ABA model and TLR4 KO in IECs, each alone, are associated with a lower plasma level of GLP1 in males, without additive or synergic effect (Tukey's post-test, *p* < 0.001 and *p* < 0.05, respectively, Figure 5D). In contrast, TLR4 KO in IEC impaired plasma PYY concentration (*p*(TLR4^IEC^^−/−^) = 0.0749 and *p*(interaction) = 0.0223, Figure 5D). Indeed, a trend toward an increase in plasma PYY was observed in male CT mice (*p* = 0.0503) that was blunted under ABA conditions.

**Figure 6.**
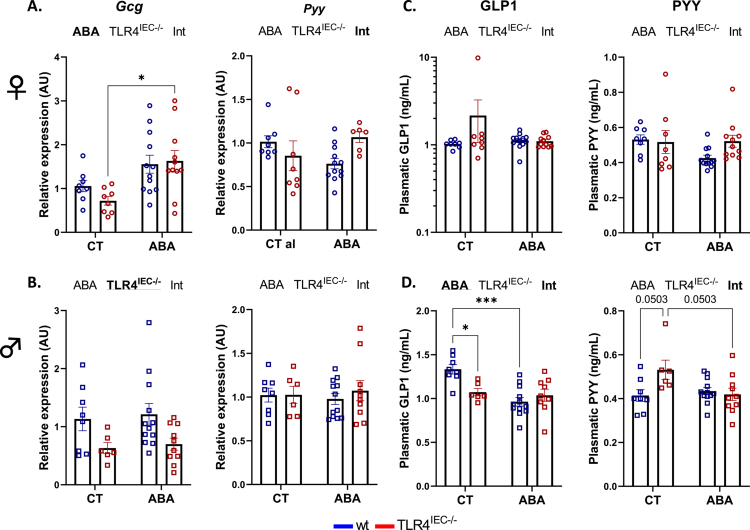
Colonic and plasmatic food intake regulation in female and male TLR4^IEC^^−/−^mice in response to the ABA model. Relative cDNA levels of the *Gcg* and *Pyy* genes in female (A) and male (B) mice. Plasmatic concentration of the anorexigenic hormones GLP1 and PYY in female (C) and male (D) mice. Wild-type (*wt*) mice (in blue) and mice invalidated for the TLR4 specifically in intestinal epithelial cells (TLR4^IEC^^−/−^, in red), were submitted to the activity-based anorexia (ABA) model or were used under control conditions (CTs). The data are shown as mean ± SEM and were analyzed using two-way ANOVA (TLR4^IEC^^−/−^ × ABA). The two-way ANOVA significance (*p* < 0.05 or ABA, TLR4^IEC^^−/−^ and/or Int for interaction) is indicated by bold and underlined font. Tukey's multiple comparisons test results are indicated as *p < 0.05, ***p < 0.001. *n* and exact *p-*value on Figure S10.

As we observed alterations of energy homeostasis in TLR4^IEC^^−/−^, we investigated the role of TMX injections in control mice (i.e. not floxed mice) to discriminate the impact of TMX alone from the impact of TLR4 KO, even if TMX injections were performed several days before the beginning of the ABA procedure. Surprisingly, TMX injections limited body weight gain, only in female mice (Figure S2A). Even if a TMX effect was significant on the kinetics of food intake in both male and female mice (*p*(TMX) < 0.05), cumulative food intake remained unchanged (Figure S2B and C). Interestingly, the eating behavior evaluated in BioDAQ food and drink intake monitor seemed to be affected by TMX alone (Figure S2C). Particularly, from day 5 to day 16, TMX-injected male mice exhibited higher quantity of pellet waste and higher number of pellets nibbled without consumption (Figure S2C) than PBS injected mice. We then compared the impact of TMX in *wt* mice to the impact of TLR4 KO in IEC under control conditions (i.e. not in the ABA model) on entero-hormones expression (GLP1 and PYY). In female mice, neither TMX injections nor TLR4 KO in IEC modified *Gcg* or *Pyy* mRNA levels or GLP1 and PYY plasmatic concentrations (Figure S3). In male mice, only TLR4 KO in IEC induced a decrease in plasma GLP1 and a trend toward an increase in plasma PYY level (Figure S3).

### The KO of TLR4 in intestinal epithelial cells affects the intestinal barrier function during activity-based anorexia model in a sex-dependent manner

To evaluate intestinal barrier function, we analyzed the expression of 19 gene markers in the colon involved in intestinal permeability (schematic overview on Figure S4C) and *Muc2* in mucus production, as well as ileal paracellular permeability (Figure 6). Again, male mice showed a more marked response compared to female mice. In females, TLR4 ^IEC^ KO in CT induced a reduction only in *Cldn1* mRNA expression (Figure 6A, gray bars) while *Ocln*, *Marveld2*, *F11r*, *Tjp1*, *Cldn7*, *Cldn12,* and *Cldn15* mRNA levels were downregulated in males (Figure 6B). None of these effects were related to TMX injections (Figure S4A and B). The ABA model *per se* induced slight effects, with an upregulation of *Cgn*, *Tjp1,* and *Cldn7* mRNA levels and a decrease in *Cldn1* mRNA expression in female mice (blue bars), while only *Tjp1* expression was downregulated in male mice. However, in male TLR4^−^^/−^ mice, ABA induced an upregulation of *Ocln*, *Marveld2*, *F11r*, *Tjp1*, *Tjp2*, *Tjp3*, *Cldn3*, *Cldn7*, *Cldn12,* and *Cldn15* mRNA expression compared to TLR4^IEC^^−/−^ control mice. In contrast, in female TLR4^IEC^^−/−^ mice, ABA was associated with an increase in *Cldn2* and *Cldn15* mRNA levels. When comparing TLR4^IEC^^−/−^ ABA mice to *wt* ABA mice, both in females and males, the mRNA expression of two factors was downregulated (*Tjp1* and *Cldn7* in females; *Cgn* and *Cldn5* in males) or upregulated (*Cldn2* and *Cldn15* in females; *Cldn3* and *Cldn7* in males). Western blots did not reveal significant differences for Cldn1, Ocln, and Tjp1 expression in females. In males, the ABA model *per se* induced an increase in Cldn1 and Ocln, which was partially blunted in TLR4^IEC^^−/−^ mice (Figure S5). We also evaluated the expression of *Muc2* mRNA, encoding the main secretory mucin, which did not show significant changes in both sexes. We only observed a trend for an impact of TLR4^IEC^ invalidation in male mice (*p*(TLR4^IEC^^−/−^) = 0.0560; Figure 6E). All these data highlight the putative role of intestinal epithelial TLR4 in the sex-specific regulation of gut barrier functions, even if we did not observe significant differences for ileal paracellular permeability (Figure 6C‒F).

### The KO of TLR4 in intestinal epithelial cells affects intestinal inflammatory response during activity-based anorexia model in a sex-dependent manner

**Figure 7.**
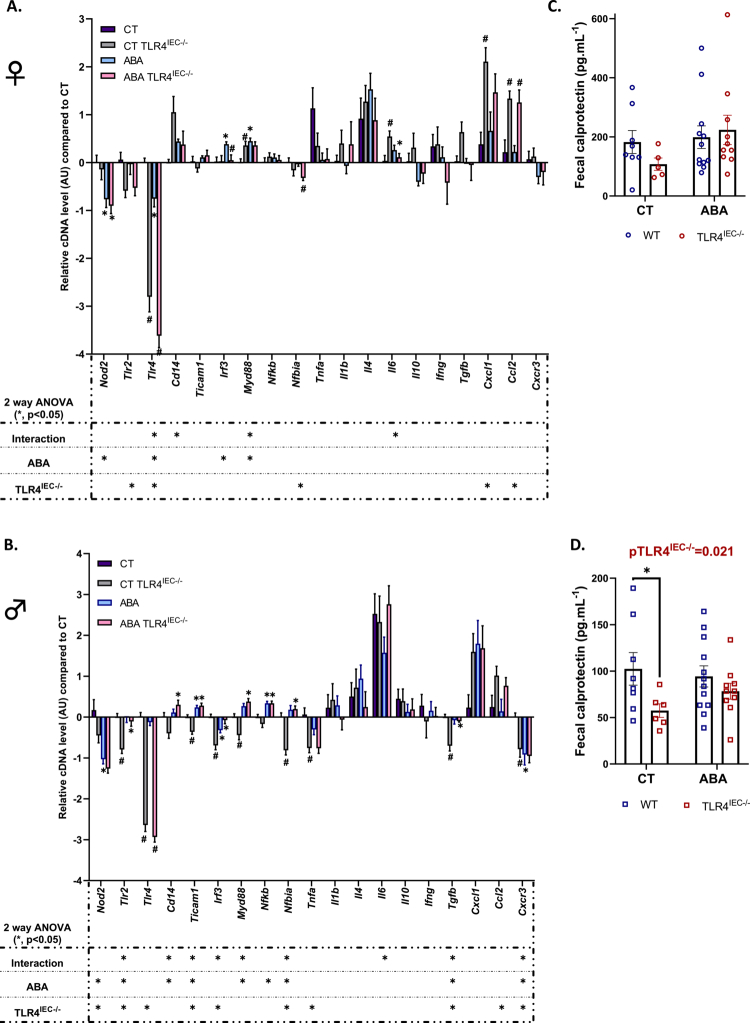
Colonic immunomodulation in female and male TLR4^IEC^^−/−^ mice in response to the ABA model. Relative cDNA levels of inflammation markers in the control (CT) TLR4^IEC^^−/−^, activity-based anorexia (ABA), and ABA TLR4^IEC^^−/−^ groups normalized to wild-type (*wt*) CT group in the proximal colon of female (A) and male (B) animals. *wt* CT mice or mice submitted to the activity-based anorexia (ABA) model were compared to mice invalidated for the TLR4 specifically in intestinal epithelial cells (TLR4^IEC^^−/−^). The data are shown as mean ± SEM bar plot and were analyzed using two-way ANOVA (TLR4^IEC^^−/−^ × ABA). The significance (*p < 0.05) was indicated just below the graph. Tukey's multiple comparisons tests are indicated as * (CT vs. ABA; CT TLR4^IEC^^−/−^vs ABA TLR4^IEC^^−/−^) or # (CT vs. CT TLR4^IEC^^−/−^; ABA vs. ABA TLR4^IEC^^−/−^). *n* and exact *p-*value on Figure S10. Fecal calprotectin in females (C) and males (D). *wt* mice (in blue) and TLR4^IEC^^−/−^ (in red) submitted to ABA or not (CT). Two-way ANOVA (TLR4^IEC^^−/−^ × ABA) and nested *t*-test *p < 0.05, encompassing duplicates values.

To evaluate intestinal inflammatory responses, we measured the expression levels of 19 gene markers in the colonic mucosa (Figure 7). Under control and ABA conditions, TLR4 KO in IEC decreased TLR4 mRNA levels in both female and male mice, as expected (Figure 7A and B). In control conditions, female TLR4^IEC^^−/−^ mice only exhibited an increase in *Myd88*, *Il6*, *Cxcl1,* and *Ccl2* mRNA expression compared to control *wt* mice (Figure 7A) whereas mRNA levels for eight inflammatory markers were downregulated in male CT TLR4^IEC^^−/−^ mice (Figure 7B), including six pro-inflammatory (*Tlr2*, *Ticam1*, *Irf3*, *Myd88*, *Tnfα*, and *Cxcr3*) and two anti-inflammatory markers (*Nfκbiα* and *Tgfβ*). Interestingly, only males showed a lower fecal calprotectin concentration after TLR4 KO in IEC (Figure 7C and D). In a reassuring manner, TMX injections in control mice did not reproduce these effects (Figure S6A and B). All of these differences disappeared in ABA conditions (ABA TLR4^IEC^^−/−^ vs. ABA *wt* mice) in both female and male mice, except for *Ccl2* in females. The ABA model *per se* also induced some sex-dependent effects on colonic inflammatory markers. A reduction in *Nod2* and *Tlr4* mRNA expression and an increase in *Irf3* and *Myd88* mRNA levels were observed in female ABA mice. In contrast, male ABA mice showed a decrease in *Nod2*, *Irf3,* and *Cxcr3* mRNA levels and an increase in *Ticam1* and *Nf-κb* mRNA expression (Figure 7A and B). The impact of the ABA model in TLR4^IEC^^−/−^ mice majored the difference according to sex. Indeed, female TLR4^IEC^^−/−^ ABA mice only exhibited a reduction in *Nod2* and *Il6* mRNA levels compared to TLR4^IEC^^−/−^ CT mice (Figure 7A), which may be explained, respectively, by an ABA or an interaction effect. When male TLR4^IEC^^−/−^ ABA mice were compared with male TLR4^IEC^^−/−^ control mice, *Tlr2*, *Cd14*, *Ticam1*, *Irf3*, *Myd88*, *Nfκb, Nfκbia,* and *Tgfβ* mRNA levels were increased (Figure 7B). However, fecal calprotectin was not statistically modified in ABA TLR4^IEC^^−/−^ mice compared to *wt* ABA mice, both in females and males (Figure 7C and D). All these data suggest that TLR4 in IEC plays a more important role in modulating colonic inflammation in males under physiological conditions. However, the ABA context for 12 d blunted this phenomenon.

**Figure 8.**
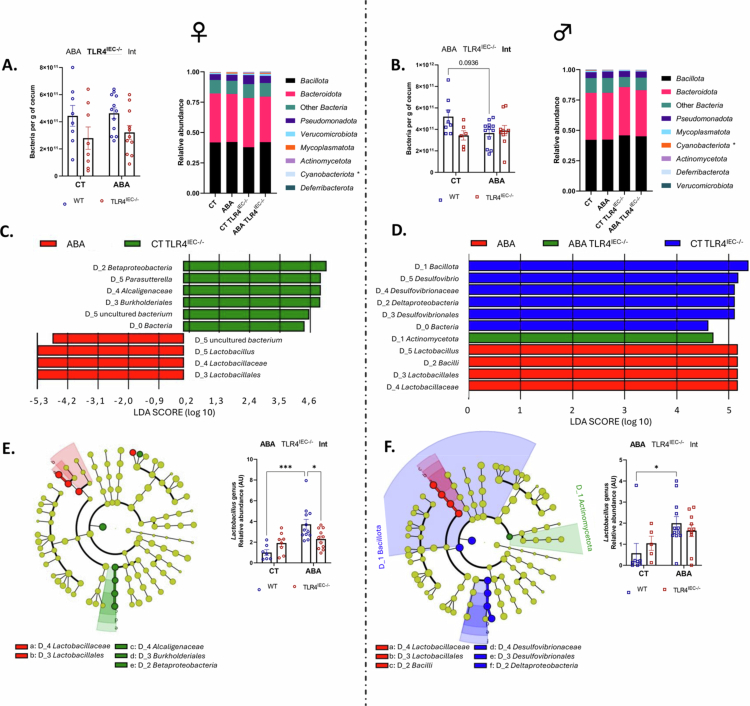
Gut microbiota composition in female and male TLR4^IEC^^−/−^mice in response to the ABA model. The gut microbiota composition of the cecal content was analyzed in female and male wild-type (*wt*) mice and in mice invalidated for the TLR4 specifically in intestinal epithelial cells (TLR4^IEC^^−/−^). The mice were under control condition (CT) or submitted to the activity-based anorexia (ABA) model. The total number of eubacteria per gram of cecal content (A and B), the relative abundance of phyla, linear discriminant analysis (LDA) (C and D), cladogram (E and F) and abundance of the *Lactobacillus* genus (E and F) are shown. (C and D), The number preceded by D_ represented the taxa level; domain-0, phylum-1, class-2, order-3, family-4, and genus-5. (A, B, E, and F). The data were analyzed by two-way ANOVA (TLR4^IEC^^−/−^ ×  ABA). The data are expressed as mean ± SEM. Significant (*p* < 0.05) results are indicated in bold and underlined font (ABA, TLR4^IEC^^−/−^ and Int for interaction). Tukey's multiple comparison test is indicated as *p < 0.05 (*n* = 5–12 per group).

### The KO of TLR4 in intestinal epithelial cells affects gut microbiota composition during activity-based anorexia model in a sex-dependent manner

We then determined the microbiota composition by performing V5–V6 16S rRNA gene sequencing on DNA extracted from the cecal contents. In female mice, we observed that the total bacterial abundance was reduced in response to TLR4 KO in IECs in both CT and ABA mice, whereas the ABA model did not affect it (Figure 8A). In contrast, in male mice, the bacterial abundance seemed to be reduced by both TLR4^IEC^^−/−^ and the ABA model without additive effects (*p*(int) < 0.05, Figure 8B); however, the differences did not reach significance. In male mice, the alpha diversity index (Pielou's evenness) differed between *wt* ABA mice and the CT group, while it remained unaffected in female mice (Figure S7). The Shannon combined index (richness and repartition) was not significantly affected by TLR4 KO and ABA model in both male and female mice (Figure S7). Concerning the beta diversity evaluated by the Jaccard distance, the statistical analysis (PERMANOVA) showed differences among the four groups in females: CT TLR4^IEC^^−/−^ vs. CT, ABA vs. CT, ABA TLR4^IEC^^−/−^ vs. ABA, and ABA TLR4^IEC^^−/−^ vs. CT TLR4^IEC^^−/−^. In males, only ABA TLR4^IEC^^−/−^ differed from CT TLR4^IEC^^−/−^ in a significant manner (Figure S7). In addition, the unweighted UniFrac method to alleviate beta diversity also did not show sex-specific differences, but this finding indicated that TLR4^IEC^^−/−^ modified the gut microbiota in CT mice (*q* < 0.05 for both female and male), which was blunted in ABA mice (Figure S7).

At the phylum level, we observed differences in relative abundances between the groups, which were more pronounced in males (Figure 8A–D). In male CT TLR4^IEC^^−/−^ mice, the abundance of the *Bacillota* phylum was increased compared to *wt* mice. Similarly, the abundance of the *Deltaproteobacteria* class was increased, especially the *Desulfovibrionales* order and its associated taxa (*Desulfovibrionaceae* family and *Desulfovibrio* genus; Figure 8D). Male ABA TLR4^IEC^^−/−^ mice showed an increase in the abundance of the *Actinomycetota* phylum (Figure 8B and D). In contrast, in female mice, CT TLR4^IEC^^−/−^ showed higher levels of *Betaproteobacteria* class (in the *Pseudomonadota* phylum) and *Burkholderiales* order, with higher levels of *Alcaligenaceae* family and *Parasutterella* genus (Figure 8A and C). In response to ABA, both sexes showed elevated levels of the *Lactobacillus* genus (Figure 8E and F). It is noteworthy that this observed difference in *Lactobacillus* abundance was no longer observed in the ABA TLR4^IEC^^−/−^ mice compared to CT TLR4^IEC^^−/−^ mice for both sexes (Figure 8E and F). Of note, the administration of TMX did not affect the total number of bacteria (Figure S8A). However, male mice that received TMX exhibited a reduction in the total number of observed ASVs and in the Shannon index, in comparison to those that received PBS. The Jaccard distance was significantly different between males injected with TMX or PBS, which was not observed for the unweighted UniFrac method (Figure S8E, PERMANOVA). The relative abundance of taxa (Figure S8B) revealed some compositional differences; however, these differences did not align with the alterations observed following TMX-induced TLR4^IEC^^−/−^. All these data highlight that TLR4 KO in IEC induced sex-specific alterations of gut microbiota composition that were more pronounced in CT mice.

## Discussion

In the present study, we provide evidence that intestinal epithelial TLR4 contributes to the regulation of energy homeostasis in response to activity-based anorexia (ABA) model in a sex-dependent manner. Of interest, we show that intestinal epithelial TLR4 KO is associated with more pronounced alterations of colonic inflammatory markers and factors involved in the regulation of gut barrier function in male compared to female mice under both control and ABA conditions. Our data also provide evidence of sex-specific alterations in the gut microbiota composition in response to intestinal epithelial TLR4 KO.

First, as we partially previously reported,^10^ intestinal epithelial TLR4 KO is associated with an initial limitation of body weight loss in response to the ABA model in both sexes. This effect was associated with a decrease in running wheel activity in female mice, despite a decrease in food intake. In contrast, in male mice, TLR4^IEC^ KO induced a slight increase in food intake, associated with a reduction of food-anticipatory physical activity. In CT mice, TLR4^IEC^^−/−^ did not affect body weight, as previously described.^10^

The TLR4 KO in intestinal epithelial cells induces a disruption of the dialog between the gut microbiota and intestinal cells, which may interfere with the role of the gut microbiota–gut–brain axis in the pathophysiology of the ABA model.^15^ For instance, it has been reported that TLR4 plays a role in mucin production. Cold-inducible RNA-binding protein induced mucin production through the TLR4/NF-κB signaling pathway.^26^ Intestinal epithelial TLR4 is essential for the proper development of goblet cells in mice.^27^ In airway epithelial cells, LPS from *Pseudomonas aeruginosa* can induce *Muc5ac* mucin gene expression via TLR4-dependent pathways.^28^ Thus, TLR4 signaling contributes to the regulation of multiple mucin genes, including *Muc5ac*, *Muc5b*, and *Muc7*, in response to various inflammatory stimuli and pathogens.^29^ Interestingly, female ABA mice exhibited *Muc5ac* alteration in the colonic mucosa^30^ but also TLR4 expression modification.^11^ TLR4^IEC^ KO induced a low anti-inflammatory environment in the colon of control male mice that was not observed in female mice. Similarly, factors involved in gut barrier functions were mainly affected in control males but not in females. The novelty of our data is the fact that we used inducible TLR4^IEC^ KO and not a constitutive KO as in previous studies. For instance, Crame et al. reported that constitutive TLR4^IEC^ KO did not impact gut barrier function and goblet cells both in ileum and colon of mice,^31^ but male and female mice were mixed. Similarly, in male mice, non-conditional intestinal TLR4 KO did not alter gut barrier function under control conditions, while a gut dysbiosis was observed, which might be due to the different origin of mice; *wt* C57BL6/J strain came from the Shanghai SLAC Laboratory Animal Co. Ltd., whereas the Villin^+/Cre^/*TLR4*^*fl/fl*^ mice were bred by the authors.^32^ In contrast, mice with intestinal epithelial KO of Myd88, a downstream factor in the TLR4 signaling cascade, showed a gut barrier disruption, an increased mucus layer, and a dysbiosis, particularly a decrease in *Lactobacillus* abundance.^33^ In our model, conditional intestinal epithelial TLR4 KO also induced a gut dysbiosis associated with alteration of gut barrier markers but did not modify *Lactobacillus* abundance. In the present study, intestinal epithelial TLR4 KO is associated with an increase in *Desulfovibrionaceae* in male but not in female mice. Interestingly, *Desulfovibrionaceae* are sulfate-reducing bacteria that produce H_2_S and are closely linked to intestinal homeostasis (mucus production and inflammation). In adapted concentration, they are reported as anti-inflammatory.^34^^,^^35^ In contrast, intestinal epithelial TLR4 KO is associated with an increase in *Parasutterellla* and *Burkholderiales* abundance only in females. *Parasutterellla* have been previously reported to contribute to lipid metabolism,^36^ and the abundance of *Burkholderiales* was shown to be reduced in a previous study on ABA mice^24^ and was associated to BMI in mice fed with western diet.^37^ A strength of our study is the use of a model with inducible TLR4^IEC^ KO, which limits long-term adaptations or compensatory mechanisms since TLR4 KO was induced in adult mice 22 d before sampling. Our results could thus reflect the early response of IECs to TLR4^IEC^^−/−^. However, further investigations should deeply evaluate the role of these TLR4^IEC^^−/−^ induced gut microbiota alterations by evaluating the effects of microbiota transplantation from TLR4^IEC^^−/−^ into naïve mice on the intestinal inflammatory response and gut barrier function.

In mice with TLR4^IEC^ KO, the ABA model induced sex-dependent effects on inflammatory markers and gut barrier actors. To our knowledge, there is no previous study evaluating the impact of inducible TLR4^IEC^ KO on gut inflammatory responses, barrier function and gut microbiota composition under pathophysiological conditions in both sexes. In a model of acute pancreatitis, constitutive TLR4^IEC^ KO exacerbated systemic inflammation, ileal permeability, and gut dysbiosis in male mice,^32^ but there was no data in females. In the context of obesity, the gut microbiota has been proposed to contribute to the sex-dependent response to a high-fat diet.^21^^,^^38^ It is thus interesting to deeply decipher the role of intestinal epithelial TLR4 KO in AN-like models and to evaluate its deleterious or beneficial impact. We can speculate that the gut microbiota is differentially affected between males and females by both intestinal TLR4 KO and ABA model mice and that this may contribute to the global sex-specific response to restricted conditions. As often described in the ABA model^18^^,^^24^ and in patients with AN,^15^ *Lactobacillus* genus was increased in *wt* ABA mice in both sexes, which was not observed in TLR4^IEC^^−/−^ mice. *Lactobacillus* are commonly considered as beneficial to limit gastrointestinal or anxiety disorders for instance, even if clinical efficacy remains to be confirmed.^39^ The increase of *Lactobacillus* in AN and in ABA mice could be an adaptive mechanism to malnutrition.^40^ Indeed, in another model associated with a malnourished status, i.e. rats with small intestine resection, *Lactobacillus* abundance is also increased.^41^ In the present study, the lack of an increased *Lactobacillus* abundance in TLR4^IEC^^−/−^ ABA mice compared to TLR4^IEC^^−/−^ CT mice might be related to the observed limitation of body weight loss. Thus, the role of *Lactobacillus* isolated from ABA mice donors should be further evaluated in recipient mice to determine whether body weight loss or recovery will be improved or not. Probiotics based on *Lactobacillus* might be a promising therapeutic strategy to restore body weight, as shown in malnourished juvenile mice.^42^ In addition, the increase of *Desulfovibrionaceae* induced by TLR4^IEC^^−/−^ in CT mice was not observed under ABA conditions. In patients with AN, *Desulfovibrionaceae* abundance has been shown increased before and after weight recovery.^43^ Only the abundance of the *Actinomycetota* phylum was specifically altered in ABA TLR4^IEC^^−/−^ mice in males but not in females. Interestingly, this alteration was associated to more colonic inflammatory and gut barrier function markers that were altered by ABA model in TLR4^IEC^^−/−^ mice. Recently, the abundance of *Actinomycetota* has been reported higher in the control group than in the non-organic anorexia group in children^44^ while the abundance of *Actinomycetota* was one of the consistent changes observed in patients with AN.^45^ Further investigations should decipher the role of the sex-specific gut microbiota alterations induced by TLR4^IEC^^−/−^ and/or the ABA model.

Our study has some limitations. Indeed, we used TMX to induce TLR4 KO, which represents a well-established technique, as evidenced by the extensive coverage in the literature.^46^ Although some TMX derivatives have been employed, it is important to note that each molecule can activate estrogen receptors.^47^ It is of interest to note that a previous study evaluated the efficacy of TMX versus 4-OH TMX, which showed comparable effects on Cre^ERT2^ activity.^48^ TMX was initially described to induce no acute toxicity or severe abnormalities in mice.^49^ However, TMX has been documented to delay total transit and alter colonic motility.^50^ Endogenous ERs are expressed not only in gonadal tissues but also in the intestine, kidney, and brain.^51^ These receptors play functional roles in the intestine and are involved in gastrointestinal disorders,^52^ which are commonly observed in AN patients, predominantly in females. It is therefore reasonable to ask to what extent the observed sex-dependent results can be attributed to the effects of TMX itself. We have thus evaluated the proper effects of TMX. Even if TMX induced some effects on body weight, eating behaviors, and the gut microbiota, it did not reproduce the impact of TLR4^IEC^ KO on inflammatory, gut barrier markers, and the gut microbiota composition in both males and females. Another point to discuss is linked to the observed results to the body weight loss. As previously reported,^8^ male mice lost more weight in response to the ABA model compared to females (approximately, 15% vs. 7%). This difference may contribute to explain the sex-dependent response to intestinal epithelial TLR4 KO to inflammatory responses and gut barrier functions in ABA mice. Further investigations in TLR4^IEC^^−/−^ mice with a similar body weight loss between male and female mice achieved by adapted caloric restriction should be of interest. In the present study, the adult male and female mice were 12 and 9 weeks old, respectively. Indeed, we chose to use male and female mice from the same litters to compare mice born, growing and living in the same environment. The corticosterone results contrast with the literature,^10^ with a decrease in this plasma hormone in response to both ABA and TLR4 KO in IECs, with a cumulative effect in females only (Figure S9A), opening a putative perspective on irritable bowel syndrome. Finally, our study did not decipher the underlying mechanisms responsible of sex-specific response to TLR4 KO in IEC and the role of sex hormones. We provided some preliminary results showing a decrease in plasma estradiol E2 and progesterone in female mice in response to TLR4^IEC^^−/−^ or to the ABA model without a cumulative effect (Figure S9B). Further investigations should decipher the role of sex hormones in the observed responses, as well as the metabolic response of TLR4^IEC^^−/−^ mice (energy expenditure, respiratory exchange ratio).

In conclusion, our study brings new elements about the role of gut microbiota‒host interactions in the pathophysiology of AN and highlights the role of intestinal TLR4 in gut homeostasis, especially in males. Inhibiting TLR4 in the intestinal epithelium would improve energy balance, which is associated with modifications of the gut microbiota, barrier function, and colonic inflammatory pathways. Antagonists of the TLR4 or Myd88 intestinal pathway should be further evaluated in the ABA model to evaluate the putative therapeutic interest, as well as the effects of probiotics. Finally, future studies should also decipher the underlying mechanisms involved in the differential response between CT and ABA mice in response to the disruption of the TLR4-mediated microbiota‒host dialog.

## Disclosure of potential conflicts of interest

The authors report there are no competing interests to declare.

## Acknowledgments

We thank Karin Varnier, Sylvie Drouet, and Yann Lacoume for their continuous help throughout the year in the housing of our OGM organisms.

We gratefully thank Sylvie Robine from the Curie Institute, Paris, France, for the gift of Villin-Cre^ERT2^ mice.

The free online DeepL tool and Biorender.com were used to improve English and figures conceptions, respectively.

## Supplementary Material

Supplementary material Fig_S1_23022026.

Supplementary material Supplemental figures.

Supplementary material Fig S10 Statistical data.

## Data Availability

The authors confirm that the data supporting the findings of this study are available within the article and its supplementary materials available online at https://doi.org/10.1101/2025.02.05.636626.

## Preprint link

https://doi.org/10.1101/2025.02.05.636626.
