# Supplementary material for: Intestinal epithelial TLR4 knock out induces sex-specific effects on gut barrier and microbiome in an activity-based anorexia model
Source: Gut Microbes. 2026 Feb 28;18(1):2637316. doi: 10.1080/19490976.2026.2637316 (PMC12959196; doi:10.1080/19490976.2026.2637316)
Supplement: Supplementary material — Fig_S1_23022026. [file KGMI_A_2637316_SM9386.docx]

# Schematic TLR4 gene of Mus musculus

**NCBI sequence identification: NC_000070.7:66745788-66765338 Mus musculus strain C57BL/6J chromosome 4, GRCm39**

|  | | | | | |  |  |  |  |
| --- | --- | --- | --- | --- | --- | --- | --- | --- | --- |
|  |  |  |  |  |  |  |  |  |  |
| **WT TLR4** | **Exon 1** | |  | **Exon 2** | |  | **Exon 3** | |  |
|  | **1** | **375** |  | **6347** | **6513** |  | **11681** | **19032** | **19551** |
|  |  |  |  |  |  | |  | | --- | | |  | | --- | |  |  |
| **Floxed TLR4** | **Exon 1** | |  | **Exon 2** | |  | **Exon 3** | |  |
|  |  |  |  |  |  | **11456** | **14025** |  |  |
|  |  |  |  |  |  | |  | | --- | |  |  |  |
| **Excised TLR4** | **Exon 1** | |  | **Exon 2** | |  |  |  |  |
|  | **1** | **375** |  | **6347** | **6513** |  |  |  |  |
|  |  |  |  |  |  |  |  |  |  |

# WT sequence with annotation of primers site and deleted sequence

Exons are in capital. Primer binding sites are in black and deleted sequence in blue.

**NCBI sequence identification: NC_000070.7:66745788-66765338 Mus musculus strain C57BL/6J chromosome 4, GRCm39**

AGCTTCCTCTTGCTGTTCCTCCAGTCGGTCAGCAAACGCCTTCTTCCTGTTCTAGTCTTCAGTCTTCTAACTTCCCTCCT

GCGACGGGGCAGATCGATTCTAGAACAAAACCAAAAGTGAGAATGCTAAGGTTGGCACTCTCACTTCCTCTTTGAATATA

GTACTTGCAGAGGGGCACCCACTGGGAGGGAAGAGGCAGGTGTCCCAGGGACTCTGCGCTGCCACCAGTTACAGATCGTC

ATGTTCTCTCATGGCCTCCACTGGTTGCAGAAAATGCCAGGATGATGCCTCCCTGGCTCCTGGCTAGGACTCTGATCATG

GCACTGTTCTTCTCCTGCCTGACACCAGGAAGCTTGAATCCCTGCATAGAGGTAtgtgtcttgatcgcatgtgatcacac

cctttcctgctagcctgccttgtttctcaaaactatccacagctcagagctccctgtgtgtgctctgcttagtttatttt

gcacgaaggagttaaactaaccaaaaacttgagaagccttggcaacaaaaagcctcagtgttaacacagggcaggaacag

gcagccaggggtgtcttgtttcatttaaggcgtctgagtcatgatttagggacttgaaattagtaaaactagtttatagt

cattgttctgtgacatacctgagagtcgttaaagaacttactgaacgtctctgaggccagtattcacgggacgaaagcat

gactgtaatcactgaaaaatgtaagtaggctgtaatttcagggctttctgtgggaactctggccactcagcttttagcgg

tcattccttccctttccaaatcaagtgaaggtagctgtgtcttttctgctgctttcgaagcatctttgagatgctttgag

tggtagctcagcaggtaaggtcagtggctgccaagcctgatgaaaatctgagttcaagcctcaagcctcacaagttagag

gcagggaatctcctcctttaagatgtcttctcacttgcaagtgtctgccttggcaggtgtgtatatgcatgagcacacac

acaaatgaataaagggaacaattgtcttaaatgaaagaatttctattaaaaaataaaacaacaaaacacacaaaaacaca

aagacttttctaagtgattttagtattctgcaactaattctaggagataaagaaatgggaggggtgagggaaggagaggg

acagagcaacttaaaacatcaattagttactgctaaggcagtaactcccgttttggtcgaatactgagtcgtgagtaatc

tgacccatgactcattcttgttttcctcctgcacagaccacgcaattatcttagaagctcacaatagaactgagcaaaca

aggaaggaattcggggtgaggtaggctcagaagctcaaaactggttcaatgagttaagatacatgacattcacatgggga

aaaatactgttaattttaaaaagttataatcacagtatcttgctttctgattcctcagttatgttggcagagatggaatt

tccaatcagtgctacactgagataaaatcccgttgctcttggtgtctggtgtgctttgtcaactctcaaagcttgcttgt

tccttctgtaagccaggtctcagggcccttggccttgtcttcaggagtgattcctgactggtttcctagttcatattcct

ttctatacccacacacagtttcttctttatttgttgttattggtccaggggcttagatttatcaaactactcctttatac

tcttaataactctttggaaccatgatggttgcttcatcctacagggccttagcactgcctaagctaactacacacaccat

catccctcacctaggtcaaggctcaccatgctaaaattatggaatccctgtatatagtttaaaacttcactgttgatcaa

attgaaaaattaagaataaatgcatcaaattagtttcaatgatttttatgcaattaaatatagttatgatgcgtgaaata

taataaaagcatcccacactaacactggctaagcactagcctcaggtctgtctccagccctatggacaggccgaggagaa

catgttctttcctttagccagggtctgtctcacccatgcctgctctgtgtctccagagctctgaaattgctcttttcacc

aggctccataagttaccatggctggctgatgccaagcacgccccacatttccaaattcctgcagctggctggggtgtact

ttttttttattagatattttctttatatacatttcaaatgccaccctgaaagttccctataccctccccccaccctgctc

ccctatccacccagtcccacttcttggccctggcgtttccctgtactggagcataaaaagtttgggcctctcttcccagt

gatggctgattaggccatcttctgctacatatgcagctagagatacgagctctgggggtactggttagttcattttggct

ggggtgtactcttgcacaccacactctaccaccatacttttctctggagcccagttgagttgccatgtgaaggaaaacac

aacacacacttggtctacaatcaacaggtaacacaatgttgggtgcagaacctagcatcctaatttttttttattagata

ttttcttaatttacatttcaaatgctatcctcacagccccctataccctcccctctgccctgctccccaacctacccact

cctgcttcctggctctgccattcccctgtactgtttttgtaaactaatctatgttaaaaatcctccgactcaggagcctc

ttgttcttgtggagacttgaggacccaggataggggaacactaggctgttaaggcaggagtgggtgtgagggtgagggag

caccctcatagaggtaggggggtgggggacggcgagggggtagggggcttgtggagggaaaactgggaagggggataaca

tttgaaatgtaaatgagtaaaataaccaaaaaacaaacaaacaaaatcctcaggtggcagatcttggaggatccaccact

tgaattgacagcctccgactatctgcaatgtgcctctaatgctctcagccatccacaaagagaccttccttactcctgcc

tccctcttcctcttcctcttcccgactcggaagtcccacctactcatctagtgattggtttcctgtaatgtttattaggg

ggaaatcctaccacatagttaagcaattacgaagataccttatgttcaatttttgatacaggaaattagacattcagcaa

catttttgttttactggacattttgatttctcctatgcgtgtttcatatttcatagctatgtgtggcttatagctgcagt

actctaatgtggagctttgatttcaggattatctttttcattttatgtagatttctctgtgaatgtctcctcaggttgat

ttttcttgattgcctcatgtacattttcccctttaccctctccatatgctctttcattgatcatatcattttgtatgttt

gtcttttatttttccaccatttattctcccctttgtgtagaataaacaagaagggagtattactgctgggtttgttagca

tgtcaccaatgcctctcagtggttaacgctaagaccctttagtacagttcctcaggttgtggtgaccttcacccataaaa

ttccttttgttgctacttcttaactataattttgttatggtgttgaacgataatgtaactatcccctatgcaggatatgt

gatatgtgatcctgtaaatggattgtttgacccttaaatgggtcaaagtccacaggttaagaaccactggcctagatcat

gataggtcttcagttgtatgtgtagtatgtgtgaaaccagtgaaagaatgacttctgaacaccatctgatgtcctcgtgt

tctgcctgtggcttctccatgacagaaggctctgccagtttgtctacatttgttcccacttgttattatttgcttatgtt

cttttctccttttgacatacatattttttcctttaccacacatttccttgatcagctttccttctgaatctagaatctgt

gtctttgcaactttcgtagttcttattcatgttcttctctgttagctggttctatgagtgcagtgccatcagaaatcatg

taacatgtattcttgtaccacccatggcctttagcagaaaaagcctactatttaacttatacgggctggtgtcccaccaa

ttacacaatatttatcattcattcatccaacaaatgtctattgagcattgagaggtcaccatgtacctttctgagccttg

aagataaatagcaaacaaaaatcatcagagcatcaatgctcatggttcaattgataaatgaaaagcatctggaaaataac

tatataggcaagagatttaccttgtcatcaaaatctgtaaaggaaacaaaagagggtgagagaagaatttctgtctgatg

ccttactctcttagatacattgccttcaaggatccgatgatgagtaccatttagggagatgtgtgtgaagaagcctgttt

atgtatgaatcttctgactatatgtgtattaccccacctcttttattttctttgtctttagaggattttttgaagattag

tataaaatacataagttgtaagtaaatgctaatatgtagcaaggaatgaatagtaaccaatgataattaacattaatatt

tatcactttaattaatgcaagctttgagataagctctgatctcatttagccctttgagaattctattgcttttaaataag

agaaaacaaaactcactgggttaagcaaagcattttgccagatgaaatcatataattatgatattacatgaaatgttatg

gtatagggttcacaataaatgtgagaaaacagataaaactagtggagattatgatagagaaaacactcaaccctgagtac

aattttctaccactggaatccatgcactataagacagcctctgatcccaggaccaaactgagaaagtcaatgaatctaag

aacaaaaataattgtcaaaaaataaggcagaatctaggaaatgtctgtatatttttattggtactctccatgtagctgta

tataatgaaaatgatgaattagaacaacaataattttacataaaagtatatacaagcatacattaacatggcttttacat

acaactagcgaggttcacagaagatattataaagtcaaaccagcacacaagcaaaactttgtcccacactcagtattctt

tagttctttgtgtagtgttaaagactcctgcacatgtgtagctgttggccttttacatctcatgtgcaggcagccatgtc

agtgaaactttatgggtgtagcttttgacattaagaatcacagtatcacagtaaagttcgtaacctttggactcataatc

tttcgtcctcctctcagtgatccctgacctgtaggtgttggagttgtattgtaagtgcttccattggcactggactccag

aattctgcattttggttggttgtgatttttttgtcgtgatctctgtttataaagtgggagaaatagtctttcccaagcaa

tagcacagcaattagttaccaaatgccaaatggccaaccctgaaaacatatacataagtaatattatacaaactgaacag

gttctacttatatatgtgggattttatttatacaatatacaatatatatatatcaacaattaatgaagcgggcaacacgg

acttgaaaaacagcaaagacaagggagtaagaaaaaaactttaagagtggaaaaggaaaagtgaagtgatataattataa

tttcaaataatagtaataaaaaagatctactctgtaccaagtggcacacaacacttgttatgaaattaaggttttcagac

ttgagagttatgtaacacctgattctattgtttctcatttaatcataattttgttgtagcagaatgttaacatattgaga

attcaggggatattttttcttcctgatatgtggaataagatgtcttgcaaatatgaagaggcagataaataaatggagaa

ggatgggtgtgataccatatccccagaatggcaggtattttgggagtccaatgttatctttgactgtatagctaatttaa

ggccagactggtctataggaaagcttgtttcaaccaaaataaatcatgaacgaatgaatgaataggtggacaatatgttg

agtggcatgtacatgtgagagttttatcaccccattattcatctttggagaggagtgggaacacacggttggaaacataa

caattgttgtgtggtatttacaggtaGTTCCTAATATTACCTACCAATGCATGGATCAGAAACTCAGCAAAGTCCCTGAT

GACATTCCTTCTTCAACCAAGAACATAGATCTGAGCTTCAACCCCTTGAAGATCTTAAAAAGCTATAGCTTCTCCAATTT

TTCAGAACTTCAGTGGCTGGATTTATCCAGGTaatgaatgagcttttatgtgatgcagaatgtgaagtagttatttttta

tatcattgcattcttggcttagaaaaccaaggtggttctaactaaacttccttctgtcatctattcagtagtgctacaac

ttgctgtaaatccttggaaaagctacttttatttaactggtttcagttggatgggccactagataagaatatctaagggc

aattctaacctctacattatttaaaacaatttcattagatatttatgaaccatgtcttatatgttgtatgtctaaactac

agaagaagaatttatagatacaaaacccatactcctaattattaagcaggataaaatcctctttaacaaataagtaagtt

aaagtcttgtccttattattgaacatacagcacaaataaaataaatgttaactaatgctaatactgttgtttataacagt

aagtaataaaatatgtgaaaataagggcaacacactgtgtcctatagaagagtgaatgttttgttatgtgtgtgagagga

tcaggaaagattttgagacatgagtacatatgtaagatacctgaaatattgaaagtagaaaagagagtagagattgaaaa

aaaaactaacttaggagggagatgtaaatgtccaagtaaaacatcaactatgggcaagaaacagttactaagattgtcct

ttctgattcagggcatcttaccatttgttggaacataaaaacttttagccagtatttcaggcgggaagctcaatatattt

tattggttaaaattgctctttgacaatttcatacatctatgtaatgcatacagctactcttaccttcacccacactgagt

tttctctgatcactgttagctctgaccccttccaaaatgtctccaacctatattcataccttcttatttattgtttgacc

cactgattttaaccaggttctctgtgtgaccatagatttagaaaaacctatctgagactagtgaggttaaccatttgata

agcaactaaaaccagtgacggtttctccccaaaaatctaaactttggcagagaagaaatgattccatggtcccctccatg

atcagtaaatatctattggcatgatcagtgcagggaaccacagcttctatgacatcagatttgcaaagtctttgtcatgt

cccacatgtccctcatgtcccacaaatccctcctctctctgtctcttggctcttacatttctatcagattcctcgtcctt

tataatccctgactcttggagagggatttgtgaatgttcattacaggggtgatcacagaactatgttttgcttcttctag

catcttgtacatctaagaatatcctcattcactactgtttactataaagggaagtgacatttgttaaggggtataaatgt

aaatatttagacagaagtctggtactatgctaatttaactaaaccacaataaccaatgccctctctgcacctcaaacatc

agggtcataggcctctctaagcaacattttttgaacaggttaacagtactagccttggacaaaaatctaatccaagaaag

ctttgttactcctaaaatagttatgccagaatttcagcactggacacatcttgcctggcaggttcatgtaatagttcatc

tgggccatagctggaagagaccagtaatgatttttccccaccagccttcatgacacctttctgctgaaagcaaatcagca

gagagaacattggttgtgcttcagcttcatgtcagtgggttgtactgatcaaggagatccttaggtgttgaagttgaacg

atgaacctcttctctaccatattcctaaagctactggaatgtttcacacatgtgtttttgttctaaaatttagagtatgg

tattaaaagtcttctgcagagcagacaatactgtaaatcattagtgaactagaaaatgtattatactctttacaggagca

tgatagatggagaattccaaaggaagaggaccacagctctgttggtggagcctgtgctttctccaacgtttagcaccatg

tgccctgttgcttgtaacttttcctgagtctctgtcttctctcctagtaaaggaaaatggtaaatctccctccatggtga

aaagttaataaatgagagattattaaaattatttagtgagtttatgagtttgaaaacatgctatcataatcactttatta

aattgtacattctacttatcccagggagatagatttgaagagaactgaggtaagcaggtaaaaaactctaaacagaataa

tctctttttaatatagagaacatagtttttcacccagtataattgagaattgatctaaagtataatgtaagataattcct

taaaggtttggagtttgtattcaggaaaaaggtaagttcctcttcccttagctcacaggatattttgcattagagcaaag

cagacaatctactcctgtgcctttctttaaaaaaaaagataattttcattatgtaatttcaaatgttgtcccttttcctg

gtttccccccctgaaaacccactatcttcaccccctccccctgctcaccaacacacccacatccacttactggccctggc

attctcttatgttggggcatagaactttcacagcaccaagggcctctcctcccattgatgaccaactaggccattctctg

ttacatatgcagctagagccatgaatcacaccatatgttttctttggttagtggtttagtcccagggagctctgggggta

ctggttagttcatattgttgttcttcctagcactgcaaaccccttcagctccttgggtactttctgtattttattcactg

gggaccctgtgctccgtccaatggatggctgtgagcatccacttctgtatttgtcaggcactggcagaccctctcaggag

acagctatatcaggcttctgtcagaaagctcttgttgatatacacaatagtgcctcaatttgatggttgtttatgggatg

gatccccaggtggcagtctctggatggtcatgccttcagtctcttctccacactttgtctcggtaactcttttcatgggt

attttgttcccacttctaaaaaggattgaagtatgcacactttggccttccttcttcttgagtttcatgtgttttttgaa

ttgtatcttgggtattctgagcttctgggctaatatccagaattaagtgcatatcatgtgtcttcttttatgactgggtt

acctcactcaggatgatgccctccaggtccattcatttgcctaagaatgtcatagattcactgtttttaatagctgcata

gtactccactgtgcaaatgtaccatattttttgtatccatttctctgttgagggacatctaggttctttcaagcatctgg

ctattataaataaaactgctatgaacatagtagagcatgtgtccttattacaaggtgaagcatcatctggatatttgcct

tggagtggtattgctggatcctcaggtagtaccatgtccaattttctgaggaaccaccaaactgatttccagagtggtta

tatcagtttacagttctgccagcaatggaagagtgttcctccttctctacatcttgcgagcatctgctgtcacttgagtt

tttgatcttagtcattctgactggtgtgaagtggaatatcagggttgttttgatttgcatttccctgatgactaaggatg

ttaaacatttttttaggtacttttcagtcattcagtattcctcagttgagaattccttctttagttctgtaccccatttt

tcaatatacacaatcataatcatatatgtatgtatatgatttggcaatagaatcctaacagaaagtggaaacttgagaaa

gaatcaaacttagttgcctcatttagaagtggaatgatagaaactcacagaaattaatgggttcccaagatcatgcagga

agaatggagagttaacatggctccatggattcctcttgcgatattctttttaacatacctctaccttttgttaaattact

aaggaataaccaaatcacagaccaaaactcttttattacctatgaatactccaaagaaaataggaaaagtgagggaaggt

aattgggttagatttggaagtgactcttttgctaaatgtatctggcatgcatctatgacaacatctgtcatgaatcactg

ttggctgcgtctgagttctgtggctagcttgtctctgtggaagctttacgtagtacagcttacatttatcttggaataaa

atttagaatatttcattgagcttgtgagtctacactattcccactcttgccatacctttatattattcttcctcagtttc

cttgttgcccttcagtcacagagactctgttgtggctcctccgtctggcatgcctgctaactactacaacttttggatcg

ctgttttcttcatatattcttcacattcgctcatattgatcattgaaatttccacttacttattctcaagtgtaatctgc

ttttatctggtgagagagggtcaattcttttgatgtgaatattcttaacccattttcttcttcttctataaagcttactc

Forward primer binding site

atgtccctaataattaacatttacctgtgataatgacagactcaaaataactagccatcatatatcagtaaagttttgta

aacatttatgccattcttgactcttgacacctatgtgtcattatatatgcctttaaaattaactttcaccagtaatttat

catgactagcaaataa**tgaccacccatattgcctatac**tcattagttgtaaaattatatctatgtctggaaaaaatgcat

aaattaatctaagactactacatatcaactgtctttatgtaccccagttatgatcttgaattgattttttctaatggatt

tgctgcctgacatag**tgtgatagtttatcatcactgtagcaagtgtgaaaatgacaaatctgcagagttcctctcctgct**

**cacaccatcatcacctgttttgctctgtacagttttctctttacaataacatggtatatcatatctgtttgtatcatagt**

**ATGGTAGGGACTGTTATGTCATTAGAAAGGGTTTTTTTTTCAGCAAAAATACATAATTGGTATCTCTTTTGCCCATAGGT**

Deleted sequence

**GTGAAATTGAAACAATTGAAGACAAGGCATGGCATGGCTTACACCACCTCTCAAACTTGATACTGACAGGAAACCCTATC**

**CAGAGTTTTTCCCCAGGAAGTTTCTCTGGACTAACAAGTTTAGAGAATCTGGTGGCTGTGGAGACAAAATTGGCCTCTCT**

**AGAAAGCTTCCCTATTGGACAGCTTATAACCTTAAAGAAACTCAATGTGGCTCACAATTTTATACATTCCTGTAAGTTAC**

**CTGCATATTTTTCCAATCTGACGAACCTAGTACATGTGGATCTTTCTTATAACTATATTCAAACTATTACTGTCAACGAC**

**TTACAGTTTCTACGTGAAAATCCACAAGTCAATCTCTCTTTAGACATGTCTTTGAACCCAATTGACTTCATTCAAGACCA**

**AGCCTTTCAGGGAATTAAGCTCCATGAACTGACTCTAAGAGGTAATTTTAATAGCTCAAATATAATGAAAACTTGCCTTC**

**AAAACCTGGCTGGTTTACACGTCCATCGGTTGATCTTGGGAGAATTTAAAGATGAAAGGAATCTGGAAATTTTTGAACCC**

**TCTATCATGGAAGGACTATGTGATGTGACCATTGATGAGTTCAGGTTAACATATACAAATGATTTTTCAGATGATATTGT**

**TAAGTTCCATTGCTTGGCGAATGTTTCTGCAATGTCTCTGGCAGGTGTATCTATAAAATATCTAGAAGATGTTCCTAAAC**

**ATTTCAAATGGCAATCCTTATCAATCATTAGATGTCAACTTAAGCAGTTTCCAACTCTGGATCTACCCTTTCTTAAAAGT**

**TTGACTTTAACTATGAACAAAGGGTCTATCAGTTTTAAAAAAGTGGCCCTACCAAGTCTCAGCTATCTAGATCTTAGTAG**

**AAATGCACTGAGCTTTAGTGGTTGCTGTTCTTATTCTGATTTGGGAACAAACAGCCTGAGACACTTAGACCTCAGCTTCA**

**ATGGTGCCATCATTATGAGTGCCAATTTCATGGGTCTAGAAGAGCTGCAGCACCTGGATTTTCAGCACTCTACTTTAAAA**

**AGGGTCACAGAATTCTCAGCGTTCTTATCCCTTGAAAAGCTACTTTACCTTGACATCTCTTATACTAACACCAAAATTGA**

**CTTCGATGGTATATTTCTTGGCTTGACCAGTCTCAACACATTAAAAATGGCTGGCAATTCTTTCAAAGACAACACCCTTT**

**CAAATGTCTTTGCAAACACAACAAACTTGACATTCCTGGATCTTTCTAAATGTCAATTGGAACAAATATCTTGGGGGGTA**

**TTTGACACCCTCCATAGACTTCAATTATTAAATATGAGTCACAACAATCTATTGTTTTTGGATTCATCCCATTATAACCA**

**GCTGTATTCCCTCAGCACTCTTGATTGCAGTTTCAATCGCATAGAGACATCTAAAGGAATACTGCAACATTTTCCAAAGA**

**GTCTAGCCTTCTTCAATCTTACTAACAATTCTGTTGCTTGTATATGTGAACATCAGAAATTCCTGCAGTGGGTCAAGGAA**

**CAGAAGCAGTTCTTGGTGAATGTTGAACAAATGACATGTGCAACACCTGTAGAGATGAATACCTCCTTAGTGTTGGATTT**

**TAATAATTCTACCTGTTATATGTACAAGACAATCATCAGTGTGTCAGTGGTCAGTGTGATTGTGGTATCCACTGTAGCAT**

**TTCTGATATACCACTTCTATTTTCACCTGATACTTATTGCTGGCTGTAAAAAGTACAGCAGAGGAGAAAGCATCTATGAT**

**GCATTTGTGATCTACTCGAGTCAGAATGAGGACTGGGTGAGAAATGAGCTGGTAAAGAATTTAGAAGAAGGAGTGCCCCG**

**CTTTCACCTCTGCCTTCACTACAGAGACTTTATTCCTGGTGTAGCCATTGCTGCCAACATCATCCAGGAAGGCTTCCACA**

**AGAGCCGGAAGGTTATTGTGGTAGTGTCTAGACACTTTATTCAGAGCCGTTGGTGTATCTTTGAATATGAGATTGCTCAA**

**ACATGGCAGTTTCTGAGCAGCCGCTCTGGCATCATCTTCATTGTCCTTGAGAAGGTTGAGAAGTCCCTGCTGAGGCAGCA**

**GGTGGAATTGTATCGCCTTCTTAGCAGAAACACCTACCTGGAATGGGAGGACAATCCTCTGGGGAGGCACATCTTCTGGA**

**GAAGACTTAAAAATGCCCTATTGGATGGAAAAGCCTCGAATCCTGAGCAAACAGCAGAGGAAGAACAAGAAACGGCAACT**

**TGGACCTGAGGAGAACAAAACTCTGGGGCCTAAACCCAGTCTGTTTGCAATTAATAAATGCTACAGCTCACCTGGGGCTC**

**TGCTATGGACCGAGAGCCCATGGA**ACACATGGCTGCTAAGCTATAGCATGGACCTTACCGGGCAGAAGGAAGTAGCACTG

ACACCTTCCTTTCCAGGGGTATGAATTACCTAACTCGGGAAAAGAAACATAATCCAGAATCTTTACCTTTAATCTGAAGG

AGAAGAGGCTAAGGCCTAGTGAGAACAGAAAGGAGAACCAGTCTTCACTGGGCCTTTTGAATACAAGCCATGTCATGTTC

TGTGTTTCAGTTGCTTTAGAAGAGTATTGATAGTTTCAACTGAACTGAACGGTTTCTTACTTTCCCTTTTTTCTACTGAA

TGCAATATTAAATAGCTCTTTTTGAGAGGTCTTCATTCCAATTTCATCTTCCATTTTATGTCATTTTCTTTTCTTTTTTT

TTTTTATCTAATTCTATAAGAAATATGATTGATACACGCTCACAGATAGCCTGGCCAATCCTAAGAATGCTATATTTATT

AAATACAATTCCTAGTATACTTTTACTTTTATAAATTCAGTTATCGTTTTTCATGCCTTGACTATAAACTAATATCATAA

ATAAGATTGTTACAGGTATGCTAAGAAGGCCCATATTTGACTATAATTTTTTAAGAAAGTATGTAAAATATACTTTGTCA

TATTGTCACTGAATGTCATTCTTAAGTTATTACCTAAGTTATGGATGTCACAGAGTCAGTGTTAAAAATAATTTGGTTGA

TAGAAATATTTTTAATCAGGAGGGAAAAGTGGAGAGGGGTGCAGGAACAGAAATCATGATTTCATCATTTATTCTTGATT

TTTCCGGAAGTTCACATAGCTGAATGACAAGACTACATATGCTGCAACTGATGTTCCTTCTCATCAAGGATACTCTCTGA

AGGACTTGAGAACATTTTGGGGAGGAAGAAAGGTCTAACATCCTTTTCCTTCATCATTCTCATTTCTGGACATGCCTTGT

GAGATGGATGAATGTTGGGAGTACACATTTCTGCTTTCACCTTATTTCAGTCAGCATGAACACTGAATATATAATGTCAT

TTCACAGTGTGTGTGTGTGTGTGTTGTGTATGTACATATATGAACCTGTACATGTGTTTAAGTTTAAAGAGAAAATAGTG

TACAGAGCAGCTCTATATTTGTGATAGGGCTTTAAATAGTTGAGCTAATTCAGAAAAGTATGGAGATTTCTTGGTAAAGG

AAACCAAAGTAGAATCATTACAAGATCTAACAATAAAAATTTTGAAACAATCCTACAAGTAAATATATTGGATTTTCTTG

TCCATTAAGACAATATTCATACTATTGAAATTATGGAAACAACCCTTGGAAGGTTAATGCATAGAGACAGAATGCTATCT

ACTTGCAGTGGAATGTGATTTGACCTTGGAGAAGAAGCAAACCTTGCTACTTGTGAGCAGATGCATAAAGGTGGAGGTTT

TTTATTGTAAGTGAAATATGCCAGGCACAGAAGGAACTGGCCTTTCAGGAACTTTTGATGACATGAGCAAAGTTAGAAAA

AATAATATGCAGAACAATAGAAGAGGAAGACAAA**AGAAAGACAGCCCTAGGATGT**ATTCTTCACAACGATTTTAAACAAT

ATGCTTGAAAGAGAATGAAGTTATTAGTATCAATTAAGATGTCTACAATTTTCATAATTCCATTCAAACTGGAACATAGC

Reverse primer binding site

CACCTAATTATTTGTCTCTTGTTAGCCAAGTGAAATAGCAGATCAAGAATCTCCCCATTTTTCTGATATAAAAACCCAAA

TTCTAATGCAGTAAATGTCTTGTCAATCAGCCAGATAGCACAGAAGAGGCAAGGCGACAGTCTGTGCCCCTTCCCTCTCA

CAGAAACTCCTGTGCACTCTAGCCCACTGCTTCAGGCTACAAGCTAGAAAAGCAAGAAGTGAAAGTGCCACAGTTCTCTA

TGTGGTTAGTGCCAGTCAGGGTCATTCAACTTAAACCATGAGTCATTAAGAAAATACATATGCATGCATGCATTAATGCA

CAGAGTAGTTTATTTATAACAACTCTTTCCATAAAGGGCTGGGGAGTTTTCAACAAAATATAAAGGAACAATTAGTTTAA

TCAAAAGAAAGAAATATAGGCAGAAGAAAGAAATGAAAGAAAGAAAGGAAAGTTTTAACTGTGTATTCCAGGTTTAATTC

TAGAGATCTTCTGGAATTTTAGAGAGTGTGACTTTTGGAGAATTCCTAAACTCATTTTCAGATTATATTACGTATGTGAC

TTGGCCTTCATCTGTCTGAGAGCTAAGAAAGAAATGAAGATCATGCATTTATTATTAGGCCATTACAAACTAATAAATAT

AAAGATAAAAGGGAGACTCTGTGGATGAGTCTCCCTCTTGGCTTTCTTATGGGTAGTCAGAGAGAAGCACTCAGTAGCCT

TATCCTTGACAACATTTTTGTCACATTTGTTTTCCCAGTCTGTAGGACAACAGCAGTCCTTATGACTAAAGTAGATTGTA

TCTTTTTTACCTAGCTTCTATTCATCTGTGTTGTCCTAGCTTCCTTTTTGAGTCTACAGCCTTTGAGAAATCACTAGAAG

TCACTGGAACCTCATGCTTTGACTTGAGGCAGTCCTCATATGTGTTCCTAGGTACTCGAGGGGTCAGTTGGGAGACTGGG

GAGCCATATCTTAACCATCAGCTTTGCTTCCTTGGTGTTGAGCATCATGCCTGACAAAGTAAGCAGACAATGCCTGTATA

CGTGAAGAAGAGGAGAATCATTAATGCATGTTTTCTTGGTGTGCTGTTGTCCTTGATACATTCCAGTTCAGAATCTAAAG

TCCTAGGGATCTTAGCTGTCAACTTAGTTTTCCCTGTCTGTCACTTTGTATGGATGATTTAAATTGCTTCTTCACTTGGT

TGCTTGACACCATGTATTCTAAAATTTTGTGGAAGGTGTGTGTTGGGGGGGGGCGTAGTTCTAACAATAGTGTTCTCTAG

TGGATACATTAAAATCATATTCAGCTAATTAATATTTGATTAAGTTTTGCATGCTATACCGATTTGATAAACATTCACAA

AATCACAGGCTTCAAGATTTTTCTTAACACATCCAAAGTACACAGGCATTAAATGGGCAAAACTAAATATCAAACTGACT

TTATTTAATAGTTTCTCTACTGTTCTCTTTTGTTTTATGTCAAGAGTTGAATGCCACTGTTCTGTATTTTTAATTATTTA

TTGTTTGCTATTGTGAGAATTCAAAGCCAGAACTTTGAGGAGCTGACAGAGGCACTGTGGCCTATGAAGACAGTTTTTGG

AGTTAACAATTTCCTTGGTAACTATGGACTATGTCTCCACACTTCAGCTCTCATATCTGATGGAATAAACTCCTTTCCAG

GAGGCTTCTACTTATGCTAATGCACCCAAGCAAACAAGGAGGCTAATAGAACCAGCTGTTTCTGTCTTTATAGCAATTTC

CCAACATTCTACACTTGAGGATTTCTTCTGTCACATGATTTTTTTCATTGGGCATTCTTTCAATCCTTCATTAAATGGCC

GAGACTTCTCACTAGACCCCAACTCAATGAAATTCTTAAGCTGCTAGCATTGAACAACACTGACTTTTTCAAAGCACCTT

GATAGGGAATTTAAGCTGGACCATCTGAAGCAGGAAAGTCTGTTGTTTTGATGGAATTTCCTAATGGTACCATTGTGGCT

TTATTTTGCCTTGTTAATGTAAGGGATTCAAAGCATTTCAACTTACTACTCATAGTTCAAGCATCTATTTTGCAGATGCA

CTGAAAATTAAGAGATTGGAGAGTTTGTCATATATATTTCCATCATCAACTATTCTAGTTCTTACTAAAGAAGGAGGGTG

CAAAAATTTGAAGGATATGTTAAAGTGCCTTCTATACTTAATGATTCTTCTAGAAAAGGCAAAGTGTTGATCTTGTTCTT

TGTTATGGTATTATATCTTCTCATGGTAATTTGAAAGAAGTTTACATACCAATTTCAGTTTGTTTACCTAGGCCTTGAGA

GTCATTCTACAGTACACGATTAGGCTACTATGAAGACAAAAGAAATCATTGTGGGGAAACTCAGTACAGCTCTAGATTTA

CCTTTTATAATAGATGAATCCCAGAATGATAAAGATCAAGCCTGGCATGATGTTAATTTAGTGGGCTAGGATCCTGGAAA

CCTCCTAAAATAGGACATCCCATGCATTTGGCCTTAGCCAGTGAGGCATCTCTGAGAAAGTGTAGAAAAACTTGCAAGGA

GGTTCAGTGCTCTGAAAGACACAGAGTCAAATGTACATGTAATTCCAGTTCTTCTTTTATATATGTGTACTTTACATAGT

CCCTGAAGTATCGAGAGGCTCAGGTATAGGTGCTACCACCTTGATAGAGTTCACTTAGCCAAAATGCAGAAATGGATGCC

CAGAGAGAATAGATTACTTGTCCTGCATCCTGTAACTTAAAATGTGTTAATAATCATCATAATAAATTCTATCTGCCAAA

TATTTCATATGTGCATGAGACTGTTTTAGTTTAATTATTAAAATTGCTTTCTGATGCAGCTCTTAGCCACATTGTCATTT

CCCATACAATGAAACTGAGACCAAAAAGCAAATTCTCCAATTCCAAGGGTAGAATTCAAGTAATCCTGATATCCAGAGCT

GCTAATTTTTTGCCACACAGTAGACTGCTGCAGTGTCTGGGCTTTTTTGCTGGGGCTCATTCACTCACTAACGGGAGAAT

CCTGTGGACAAGGTCAGCAACTCCCTTACCATCTAGAAATTGAAGGTTTCAAAGGCACTGCATGTGACTTTCCTTGATTT

CTATGGAAATGAAGATGGTCCCTCCTGTGACAGTGCTAAGTGCCGAGTCTGAGTGTAAATGTGCTTTTTGGCACAAATTG

TTCTGTTCTAATAGTGTTGATTATAATTATAAAATAATGTGTTTCTGAAAGGCTGCAAGCAATTCTGGGAATGACAATAA

GGGTTTCGAAACAACATGGTATTTATGTGAGAAGTGTTTTGTTGAAAATTAAACCTGTGTTTAGGAGAAAGgatcctgtt

gtttgctcctaagaaactatcacaccatgtaattaaatcagagccagttggttgccaattggagttcttgtctcacatga

acaatattgtatcacctacaacaaacaagatatgactgaccagaggtagccaagactctttacccaaatcctgtttctct

atcttctcagggcccagaaaaaagatggaaatgcatggtcagtttttttccaaggctgggaattaaccttgtagggtgaa

gccttcctcaagttcatctcagattgtccgtaaggaataggtttttcattcaagggccttttataggaggctgtatctgt

aaataagtgaggaattcaatgtttgagaggctgtcttgacttcctttcttgggaggaaaaacaaaatccttctatgaaga

ttaggaatgtcttcgatgttctcagacctcaaaggcagaaaaaagtatgcagtgtaatttgtttgtatgtatctctctta

aaataatatctaccataacattgtctcccaa

# Excised site after Cre activity (1768 bp)

Primers binding sites are in bold black, palindromic sequences of LoxP sites in grey and inserted bases in blue.

**tgaccacccatattgcctatac**tcattagttgtaaaattatatctatgtctggaaaaaatgcataaattaatctaagactactacatatcaactgtctttatgtaccccagttatgatcttgaattgattttttctaatggatttgctgcctgacatag**atctataacttcgtatagcatacattatacgaagttattcgatatcaagcttatcgat**acacatggctgctaagctatagcatggaccttaccgggcagaaggaagtagcactgacaccttcctttccaggggtatgaattacctaactcgggaaaagaaacataatccagaatctttacctttaatctgaaggagaagaggctaaggcctagtgagaacagaaaggagaaccagtcttcactgggccttttgaatacaagccatgtcatgttctgtgtttcagttgctttagaagagtattgatagtttcaactgaactgaacggtttcttactttcccttttttctactgaatgcaatattaaatagctctttttgagaggtcttcattccaatttcatcttccattttatgtcattttcttttcttttttttttttatctaattctataagaaatatgattgatacacgctcacagatagcctggccaatcctaagaatgctatatttattaaatacaattcctagtatacttttacttttataaattcagttatcgtttttcatgccttgactataaactaatatcataaataagattgttacaggtatgctaagaaggcccatatttgactataattttttaagaaagtatgtaaaatatactttgtcatattgtcactgaatgtcattcttaagttattacctaagttatggatgtcacagagtcagtgttaaaaataatttggttgatagaaatatttttaatcaggagggaaaagtggagaggggtgcaggaacagaaatcatgatttcatcatttattcttgatttttccggaagttcacatagctgaatgacaagactacatatgctgcaactgatgttccttctcatcaaggatactctctgaaggacttgagaacattttggggaggaagaaaggtctaacatccttttccttcatcattctcatttctggacatgccttgtgagatggatgaatgttgggagtacacatttctgctttcaccttatttcagtcagcatgaacactgaatatataatgtcatttcacagtgtgtgtgtgtgtgtgttgtgtatgtacatatatgaacctgtacatgtgtttaagtttaaagagaaaatagtgtacagagcagctctatatttgtgatagggctttaaatagttgagctaattcagaaaagtatggagatttcttggtaaaggaaaccaaagtagaatcattacaagatctaacaataaaaattttgaaacaatcctacaagtaaatatattggattttcttgtccattaagacaatattcatactattgaaattatggaaacaacccttggaaggttaatgcatagagacagaatgctatctacttgcagtggaatgtgatttgaccttggagaagaagcaaaccttgctacttgtgagcagatgcataaaggtggaggttttttattgtaagtgaaatatgccaggcacagaaggaactggcctttcaggaacttttgatgacatgagcaaagttagaaaaaataatatgcagaacaatagaagaggaagacaaa**agaaagacagccctaggatgt**

**
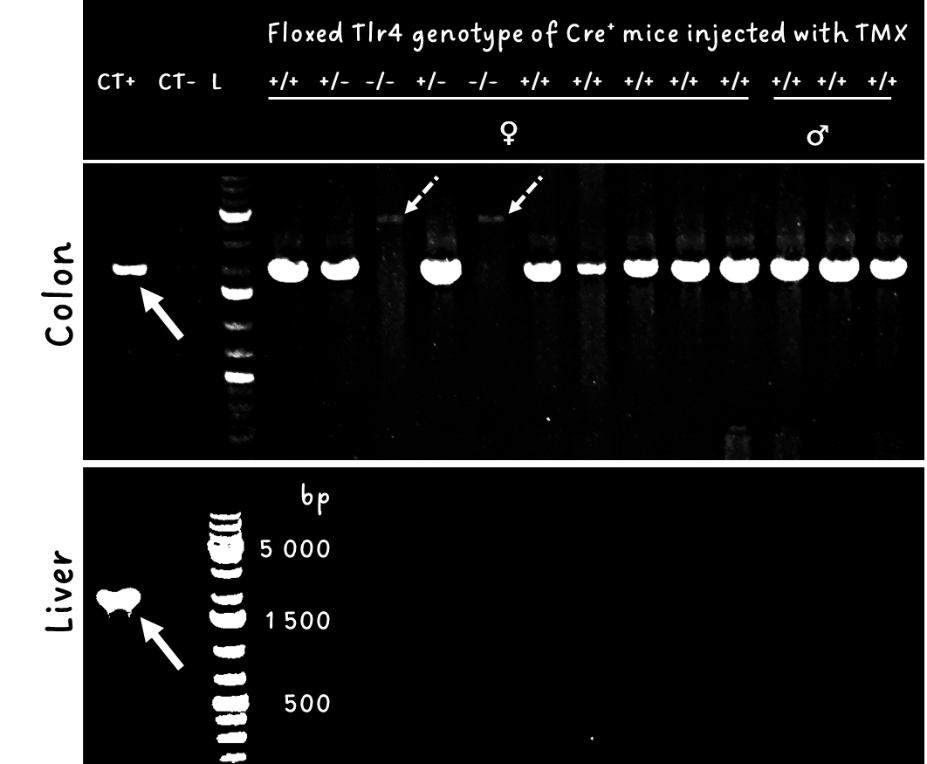
**

**Cre activity detection**

Villin specificity, Cre activity detection and impact of floxed genotype (homo or heterozygote) were evaluated by agarose 1% gel electrophoresis after PCR amplification.

Full arrows indicate excised site/Cre activity (1 768 bp) and slight dotted arrows indicated the full sequence (4 279 bp) of non floxed TLR4 sequence.

# Specificity of villin conditional Cre expression

**Table of Cre activity signal in different tissues and mice genotype, 17 days after 5 daily 1 mg tamoxifen injections.**

**Fig.S1 – Description of LoxP sites of floxed TLR4 mice**

(A) Genetic structure of *Tlr4* gene and LoxP sites before and after Cre activation. (B) DNA sequence of *Tlr4* *mus musculus* gene (S1B) with exons (in capital letters) and introns (in lower-case letters), oligonucleotides used for genotyping (in black) and deleted sequence by Cre (in blue). (C) Analysis of *Tlr4* gene recombination triggered by the Cre recombinase. (D) Evaluation of gut specificity and efficiency of Cre activity on 13 independent animals.
