# Supplementary material for: Intestinal epithelial TLR4 knock out induces sex-specific effects on gut barrier and microbiome in an activity-based anorexia model
Source: Gut Microbes. 2026 Feb 28;18(1):2637316. doi: 10.1080/19490976.2026.2637316 (PMC12959196; doi:10.1080/19490976.2026.2637316)
Supplement: Supplementary material — Supplemental figures. [file KGMI_A_2637316_SM5456.docx]

## Supplemental figures captions


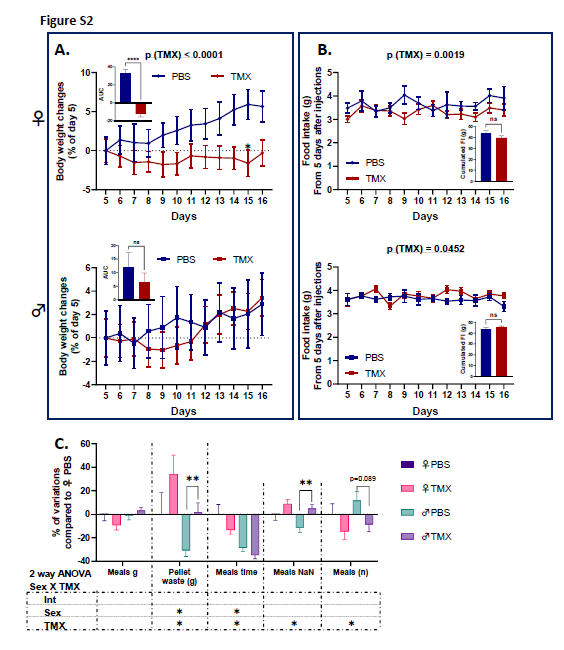


**Fig.S2 – Tamoxifen impact on body weight and food intake from day 5 to 16 in non floxed animals.**

Body weight changes (A) and food intake in g (B) in female (circles) and male (squares). C57Bl/6 wild type mice injected with PBS (in blue) or tamoxifen (TMX, in red). Data are shown as mean ± SEM and analyzed using two-way ANOVA (TMX X time). The significant p value (TMX) is indicated in each graph. Šídák's multiple comparisons test are indicated as *p<0.05 for TMX vs PBS. Area under the curve (A) and cumulated food intake (B) are shown in each panel with ****p<0.0001 (t-test). Eating behavior (C) was assessed in BioDAQ food and drink intake monitor. Percent of variations of meals quantity, powder produced, time per meal, the number of pellets nibbling without food consumption (meals NaN) and the number of meals were plotted with mean ± SEM and analyzed using two-way ANOVA (Sex X TMX), *p<0.05. Mann-Whitney was used to compare specifically TMX groups compared to their respective PBS group, **p<0.01. n=6-10 per group.


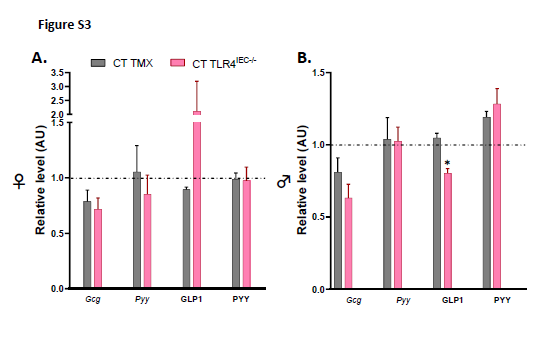


**Fig.S3 – Colonic and plasmatic food intake regulation in female and male is not affected by tamoxifen.**

cDNA relative levels were studied for *Gcg* and *Pyy* genes using the 2^-ΔΔCt^ method and plasmatic concentration of anorexigenic hormones GLP1 and PYY were evaluated by ELISA in female (A) and male (B) mice. C57Bl/6 mice (CT TMX in grey) or CreVillin^ERT2^ TLR4_floxed_ mice (CT TLR4^IEC-/-^ in red) were injected by tamoxifen (TMX), both under *ad libitum* conditions. Data are normalized on their respective controls injected with PBS (CT, dotted line) and shown as bar plots with means ± SEM, and analyzed using Kruskal-Wallis followed by Dunn’s multiple comparisons test, *p<0.05. n=6-10 per group.


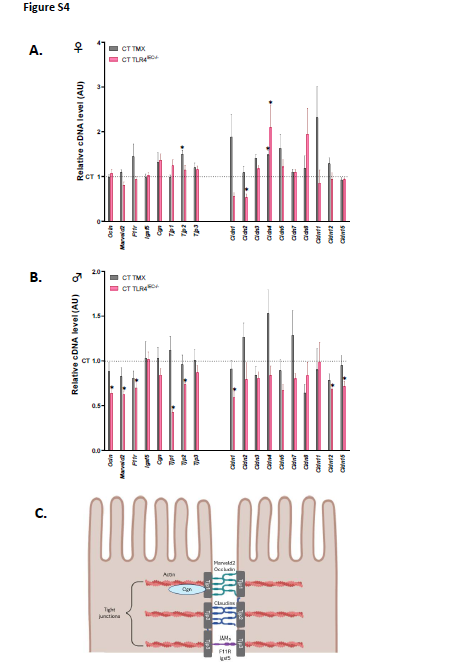


**Fig.S4 – Colonic permeability evaluated by RT-qPCR in female and male is not strongly affected by tamoxifen.**

Evaluation of molecular dysfunctions in gut permeability of proximal colon of (A) females and (B) males control mice (CT) to differentiate tamoxifen (TMX) effects alone from invalidation of TLR4 in intestinal epithelial cells (CT TMX and CT TLR4^IEC-/-^ respectively). Kruskal-Wallis followed by Dunn’s multiple comparisons were used here. Significant (p<0.05) test in response to TMX injections, in wild type mice (CT TMX) or mice expressing Cre recombinase (CT TLR4^IEC-/-^) compared to respective control injected with PBS are indicated. Data are expressed as mean ± SEM. n=4-10 /group. (C) Key components and functions of epithelial tight junctions. Tight junctions (TJs) are multiprotein complexes composed of three major transmembrane protein family that regulate paracellular permeability and epithelial polarity. Claudins form the 'suture' and determine ion selectivity. Claudins that reinforce the gut barrier (e.g. claudin-1, -3, -4, -5 and -7) restrict solute flux, whereas pore-forming claudins (e.g. claudin-2, -10 and -15) permit the selective passage of cations or water. Claudin-8, -11 and -12 contribute to the specialisation of tissue barriers. Occludin and Marveld2 stabilise TJ strands and modulates barrier signalling. Junctional adhesion molecules (JAMs), as well as the related F11R (JAM-A) and IGSF5 (JAM4), mediate homophilic adhesion and leukocyte trafficking. Cytoplasmic scaffolds (TJP1–3, ZO-1, ZO-2 and ZO-3) anchor transmembrane components to the cortical actin cytoskeleton and recruit regulatory proteins. Cingulin connects actin filaments with TJPs, thereby maintaining junctional tension and integrity. This figure was adapted from Mohammad and Thiemermann, 2021 [53].


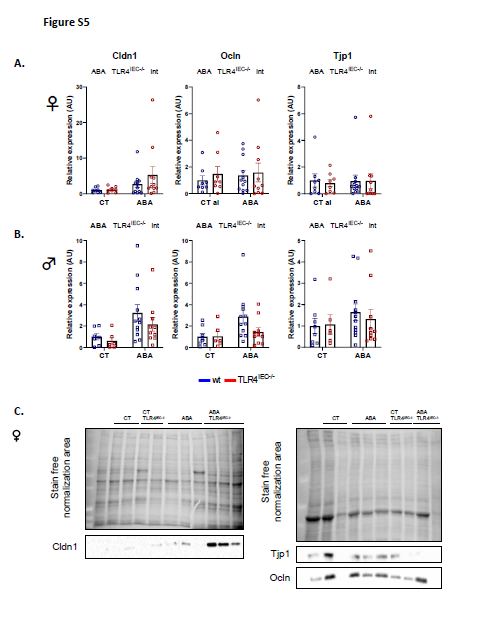


**Fig.S5 – Colonic gut permeability in female and male TLR4^IEC-/-^ mice in response to the ABA model: western blot.**

Relative proteins levels of tight junction protein markers in control (CT) TLR4^IEC-/-^, activity-based anorexia (ABA) and ABA TLR4^IEC-/-^ groups normalized to wild type (*wt*) CT group in proximal colon of female (A) and male (B) animals. *wt* CT mice or mice submitted to the activity-based anorexia (ABA) model were compared to mice invalidated for the TLR4 specifically in the intestinal epithelial cells (TLR4^IEC-/-^). Data are shown as mean ± SEM bar plot and analyzed using two-way ANOVA (TLR4^IEC-/-^ X ABA). The significance (*p<0.05) were indicated in bold and underlined. Tukey’s multiple comparisons tests did not reveal significant differences.

**
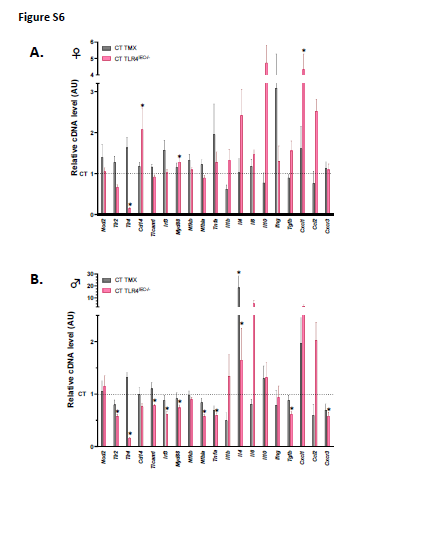
**

**Fig.S6 – Colonic immunomodulation in female and male is not strongly affected by tamoxifen.**

Evaluation of molecular dysfunctions in immunomodulation of proximal colon of (A) females and (B) males control mice (CT) to differentiate tamoxifen (TMX) effects alone from invalidation of TLR4 in intestinal epithelial cells (CT TMX and CT TLR4^IEC-/-^ respectively). Kruskal-Wallis followed by Dunn’s multiple comparisons were used here. Significant (p<0.05) test in response to TMX injections, in wild type mice (CT TMX) or mice which expressed Cre recombinase (CT TLR4^IEC-/-^) compared to respective control injected with PBS are indicated. Data are expressed as mean ± SEM.


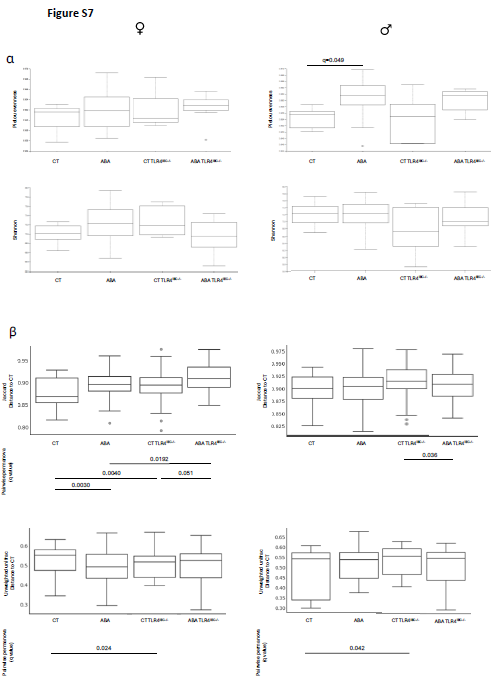


**Fig.S7 – Gut microbiota diversity indexes in female and male TLR4^IEC-/-^ mice in response to the ABA model.**

Alpha diversity (Pielou evenness and Shannon) and Beta diversity (Jaccard distance to CT) were analyzed in female and male wild type (*wt*) mice, and in mice invalidated for the TLR4 specifically in the intestinal epithelial cells (TLR4^IEC-/-^). Mice were under control condition (CT) or submitted to the activity-based anorexia (ABA) model. Data were analyzed using Kruskal Wallis or Permanova pairwise comparisons, respectively.


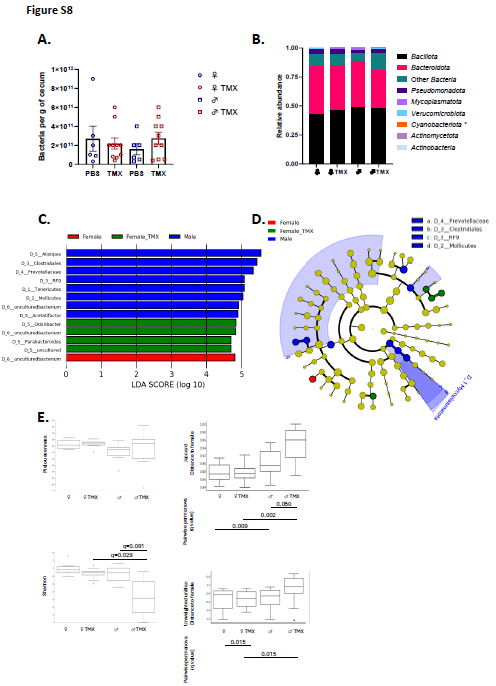


**Fig.S8 – Gut microbiota composition in female and male in response to tamoxifen (TMX).**

Gut microbiota composition in cecal content was analyzed in female and male wild type mice in response to tamoxifen (TMX) injections. The total number of eubacteria per gram of cecal content (A), the relative abundance of phyla (B), the linear discriminant analysis (LDA) score (C), cladogram (D), alpha diversity (Pielou evenness and Shannon) and Beta diversity (Jaccard distance) (E) are shown. (C), The number preceded by D_ represented the taxa level; domain-0, phylum-1, class-2, order-3, family-4 and genus-5. Data were analyzed using Kruskal Wallis or Permanova pairwise comparisons respectively (E).


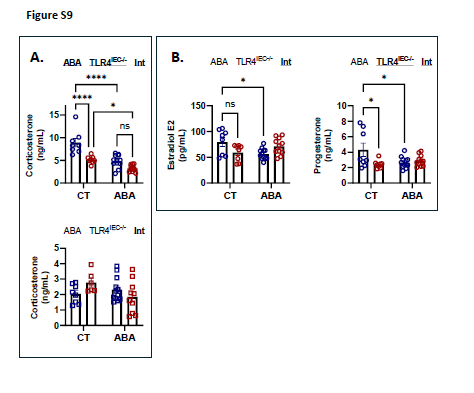


**Fig.S9 – Plasmatic levels of corticosterone and female hormones in TLR4^IEC-/-^ mice in response to the ABA model.**

The plasmatic concentrations of the stress hormone corticosterone (A) are shown for females (open circles) and males (open squares).

The plasmatic concentrations of estradiol E2 and progesterone (B) are shown for females.

Wild-type (*wt*) mice (in blue) and mice invalidated for the TLR4 specifically in the intestinal epithelial cells (TLR4^IEC-/-^, in red) were submitted to the activity-based anorexia (ABA) model or used under control conditions (CT). Data are shown as mean ± SEM and analyzed using two-way ANOVA (TLR4^IEC-/-^ X ABA). The two-way ANOVA significance (p<0.05 or ABA, TLR4^IEC-/-^ and/or Int for interaction) is indicated by bold and underlined font. Tukey’s multiple comparisons test are indicated as *p<0.05, ***p<0.001.
